# Uncovering the isoform-resolution kinetic landscape of nonsense-mediated mRNA decay with EZbakR

**DOI:** 10.1186/s13059-026-04204-w

**Published:** 2026-07-15

**Authors:** Justin W. Mabin, Isaac W. Vock, Martin Machyna, Nazmul Haque, Poonam Thakran, Alexandra Zhang, Ganesha Rai, Isabelle Nathalie-Marie Leibler, James Inglese, Matthew D. Simon, J. Robert Hogg

**Affiliations:** 1 Biochemistry and Biophysics Center, National Heart, Lung, and Blood Institute, National Institutes of Health, Bethesda, MD 20892 USA; 2 Department of Molecular Biophysics and Biochemistry, Yale University, New Haven, CT 06520 USA; 3 Institute of Biomolecular Design and Discovery, Yale University, West Haven, CT 06516 USA; 4 National Center for Advancing Translational Sciences, National Institutes of Health, Rockville, MD 20850 USA; 5 Metabolic Medicine Branch, National Human Genome Research Institute, National Institutes of Health, Bethesda, MD 20892 USA; 6 Present address: Paul-Ehrlich-Institut, Host-Pathogen-Interactions, 63225 Langen, Germany

**Keywords:** NR-seq, EZbakR, Nonsense-mediated mRNA decay, RNA quality control, Gene expression regulation, RNA kinetics, Translation termination, SMG1i

## Abstract

Cellular RNA abundance reflects synthesis and decay rates, which can differ among transcripts of the same gene. Understanding nonsense-mediated mRNA decay and other RNA turnover pathways requires isoform-resolved kinetic measurements, but existing bioinformatic tools cannot robustly estimate isoform-specific degradation rate constants. We extend the EZbakR-suite to infer isoform-level kinetics from nucleotide-recoding RNA-seq data, uncovering unexpected variability in nonsense-mediated decay efficiency among transcripts with premature termination codons and rapid decay of select mRNAs lacking premature termination codons. Our findings highlight the competition between nonsense-mediated decay and other decay pathways and provide mechanistic insights into transcript features promoting efficient decay.

**Supplementary Information:**

The online version contains supplementary material available at https://doi.org/10.1186/s13059-026-04204-w.

## Background

Transcript isoform abundance reflects the balance between the rates of RNA synthesis and degradation. Alternative splicing is a well-known mechanism for modulating isoform expression; however, the degree to which differential turnover kinetics contribute to isoform-level regulation remains less explored.

A prominent example of degradation-driven transcript isoform regulation is nonsense-mediated mRNA decay (NMD). NMD is a conserved translation-dependent RNA turnover mechanism with essential quality control and regulatory functions. The NMD pathway recognizes and degrades mRNA isoforms containing premature translation termination codons (PTCs), which can arise from genetic mutations or errors in mRNA biogenesis [1, 2]. Consequently, NMD is a central modulator of the phenotypic consequences of nonsense mutations in cancers and genetic disease [3]. Beyond quality control, NMD plays a major role in cellular gene expression regulation. Endogenous NMD substrates are frequently PTC-containing transcripts generated by alternative splicing [4], but they also include mRNAs with upstream open reading frames (uORFs) or long 3′ untranslated regions (3′UTRs) [5].

Much is still uncertain about the features that determine whether and how efficiently a transcript is degraded by NMD. Here we define NMD efficiency as the extent to which NMD reduces the abundance or stability of an NMD-targeted transcript relative to its level when the pathway is inhibited. The presence of an intron more than 50 nucleotides downstream of the termination codon is a strong predictor of NMD susceptibility [6]. Transcripts violating this rule are detected by NMD due to the persistence of exon junction complexes (EJCs) on their 3′UTRs, which enhances NMD factor association [7, 8]. Despite the utility of the 50-nucleotide rule, current models struggle to explain variable decay of transcripts predicted to contain PTCs. Studies of PTCs in paired genomic and transcriptomic datasets from tumors and healthy tissues have identified correlates of low NMD efficiency, such as a short distance between the mRNA 5′ end and the PTC or a long distance between the PTC and the next downstream exon junction [9–11]. Still, a substantial fraction (≥ 30%) of variability in decay of putative PTC-containing mRNAs is unexplained [12], with even greater uncertainty surrounding how decay specificity and efficiency are determined for NMD targets lacking PTCs [13].

The lack of effective methods for quantifying the turnover kinetics of individual transcript isoforms has been an important technical barrier to investigations of NMD. Consequently, most studies of NMD have instead relied on differential transcript abundance to infer NMD isoform sensitivity, which can be confounded by indirect transcriptional upregulation in response to NMD perturbation. In addition, transcriptome-wide measurements of transcript degradation are required to understand how competition between NMD and other decay pathways affects quality control and gene expression regulation. Therefore, new strategies are needed to directly probe isoform degradation kinetics.

Nucleotide-recoding RNA-seq (NR-seq) methods such as TimeLapse-seq or SLAM-seq provide a promising experimental route by which to overcome this challenge [14, 15]. NR-seq uses metabolic labeling and nucleotide recoding chemistry to probe the synthesis and degradation kinetics of mRNAs without the side-effects of global transcription inhibition [14–16]. NR-seq also does not require biochemical enrichment of labeled RNA, circumventing challenges of unlabeled RNA contamination and normalization across input and enrichment samples [17]. However, existing bioinformatic tools are not equipped to perform isoform-resolved kinetic parameter estimation from short-read NR-seq. We have recently developed the EZbakR-suite, which improves and generalizes many aspects of NR-seq data processing and analysis [18]. In particular, the EZbakR-suite includes a pipeline (fastq2EZbakR) for flexibly assigning sequencing reads to any transcriptome feature set of interest, enabling accurate estimation of transcript isoform kinetic parameters from NR-seq data.

Here, we extend the functionality of the EZbakR-suite by implementing a novel method to infer isoform-specific kinetic parameters. Analyses of simulated and real data confirm that this approach provides accurate estimates and captures well-established features of RNA turnover dynamics. We further implement a strategy for refinement and filtering of annotations, which we find to be important for accurate quantification of transcript degradation rate constants. To showcase the power of this approach, we use the EZbakR-suite in combination with acute NMD inhibition to map the kinetic landscape of human NMD. We quantify transcript degradation rate constants in the presence and absence of a functional NMD pathway, revealing the influence of NMD on global mRNA decay kinetics. Our data identify previously unappreciated variability in the decay kinetics of transcripts containing highly conserved poison exons and highlight the influence of competing RNA decay pathways on both the regulatory and quality control functions of NMD. We additionally identify a set of target mRNAs that lack PTCs but nevertheless are subject to highly efficient NMD. Together, our data provide mechanistic explanations for previously identified correlates of NMD efficiency, identify transcript features associated with efficient decay, and provide a roadmap for future studies of transcript isoform turnover regulation. This work illustrates how isoform-level analyses and the EZbakR-suite can be used to deepen our understanding of RNA kinetic regulation.

## Results

### Transcript isoform kinetic parameter estimation

**Fig. 1:**
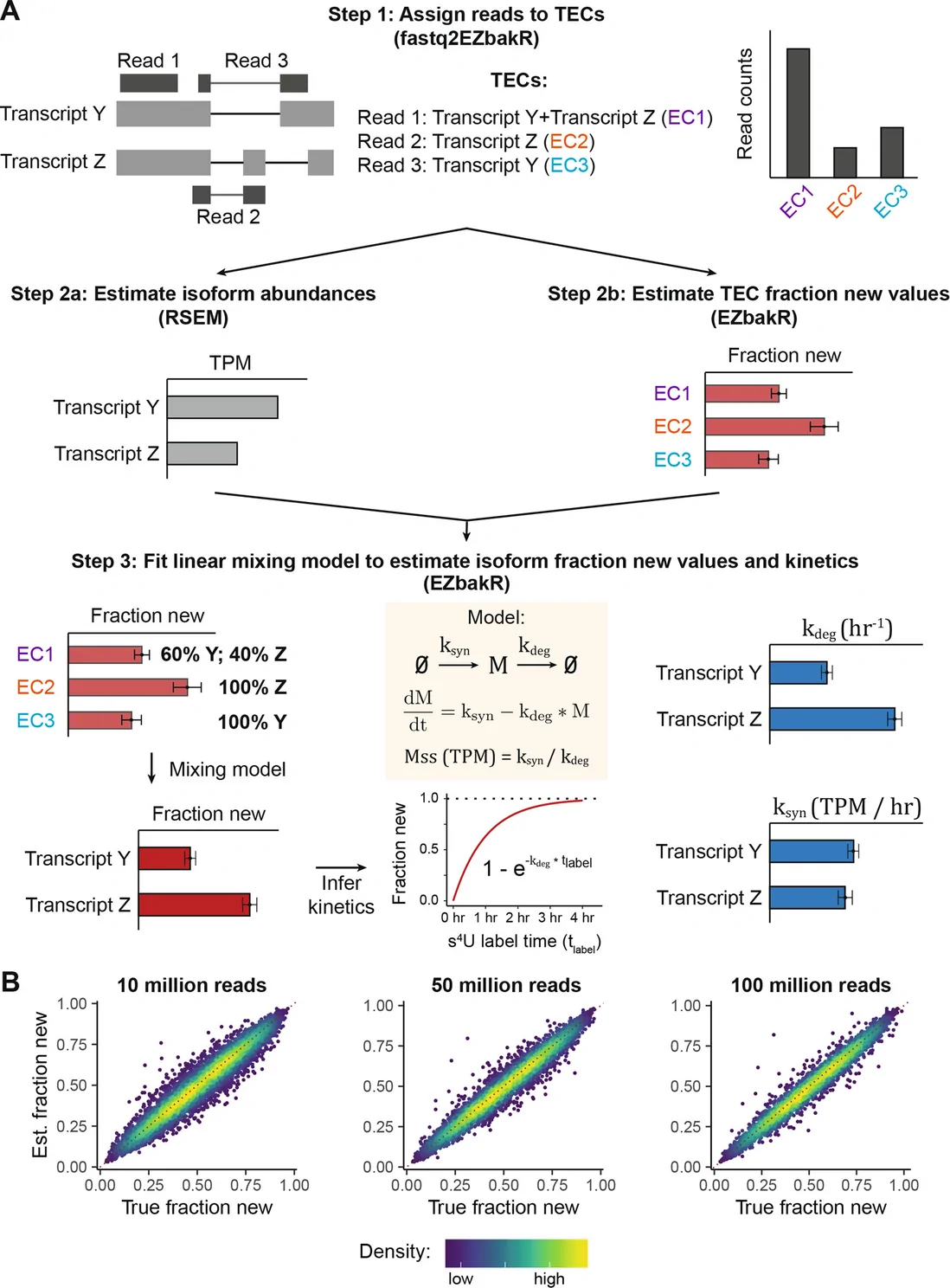
Isoform-level NR-seq analyses.** A** Schematic of the EZbakR-suite’s isoform analysis pipeline. Top: fastq2EZbakR parses transcriptome alignment files generated by STAR to assign sequencing reads to their transcript equivalence class (TEC; transcript isoforms with which a read is compatible). EC1/2/3 are the three possible TECs for the toy annotation shown on the left. Middle: Transcript isoform quantification is performed by RSEM within fastq2EZbakR, and the TEC fraction of reads from RNAs synthesized in the presence of metabolic label (TEC fraction new) is estimated by EZbakR. Bottom: Isoform abundance estimates are used to infer the fraction of reads from a specific isoform that are labeled (i.e., isoform fraction new estimates) from the TEC fraction new estimates via a linear mixing model. Isoform fraction new estimates and abundances are converted to synthesis and degradation rate constants via a standard kinetic model of NR-seq data. **B** Simulated data assessment of isoform fraction new estimate accuracy across a range of read depths. Points are colored by density, and the red dotted line represents perfect estimation (y = x)

RNA biogenesis and decay pathways often differentially affect transcript isoforms produced from the same gene. Accurate studies of RNA synthesis and decay mechanisms therefore require the ability to measure isoform-level kinetics. To meet this need, we developed a strategy to infer transcript isoform synthesis and degradation rate constants from short-read NR-seq data (Fig. 1A). This strategy is implemented as a part of our recently released EZbakR-suite, which includes a Snakemake preprocessing pipeline (fastq2EZbakR) and an NR-seq analysis R package (EZbakR) [18].

NR-seq allows identification of reads derived from transcripts produced during a metabolic labeling period, and the ratio of labeled (new) reads to total reads (the fraction new) can then be used to infer kinetic parameters [14, 19]. In our strategy for isoform-level NR-seq analysis, fastq2EZbakR first implements transcript isoform quantification using existing tools like RSEM, which assign reads to transcript equivalence classes (TECs; the set of transcript isoforms with which a read is compatible; Fig. 1A, top). EZbakR then estimates the fraction of reads from a given TEC that come from labeled RNA (i.e., the TEC fraction new; Fig. 1A, middle) [20].

We have added a novel linear mixing model to EZbakR, which combines the isoform abundance and TEC fraction new estimates to accurately infer the fraction of transcript isoforms that are labeled (i.e., the transcript isoform fraction new) (Fig. 1A, bottom). Intuitively, the model deconvolves the signal by using the measured amount of labeling in each TEC, together with the estimated isoform proportions within that TEC, to infer the extent to which transcripts from each individual isoform were labeled. From this, transcript isoform degradation rate constants are inferred as described previously [14, 19] (Fig. 1A, bottom; see also Methods). Transcript isoform degradation rate constant estimates and abundance estimates can then be combined to infer synthesis rate constants. Analyses of simulated data confirmed that EZbakR is able to accurately infer isoform fraction new values (and thus kinetic parameters) across a range of sequencing depths, read lengths, and metabolic labeling efficiencies (Fig. 1B and Additional file 1: Fig. S1). EZbakR’s isoform-level analysis also provides consistent kinetic parameter estimates across replicates and in comparisons with previously published HEK-293 and K562 RNA turnover datasets [21–24] (Additional file 1: Fig. S2A). EZbakR is thus the first tool able to extract isoform-level kinetic insights from short-read total RNA NR-seq data.

### Isoform-level analyses capture signal missed by gene-level analyses

**Fig. 2:**
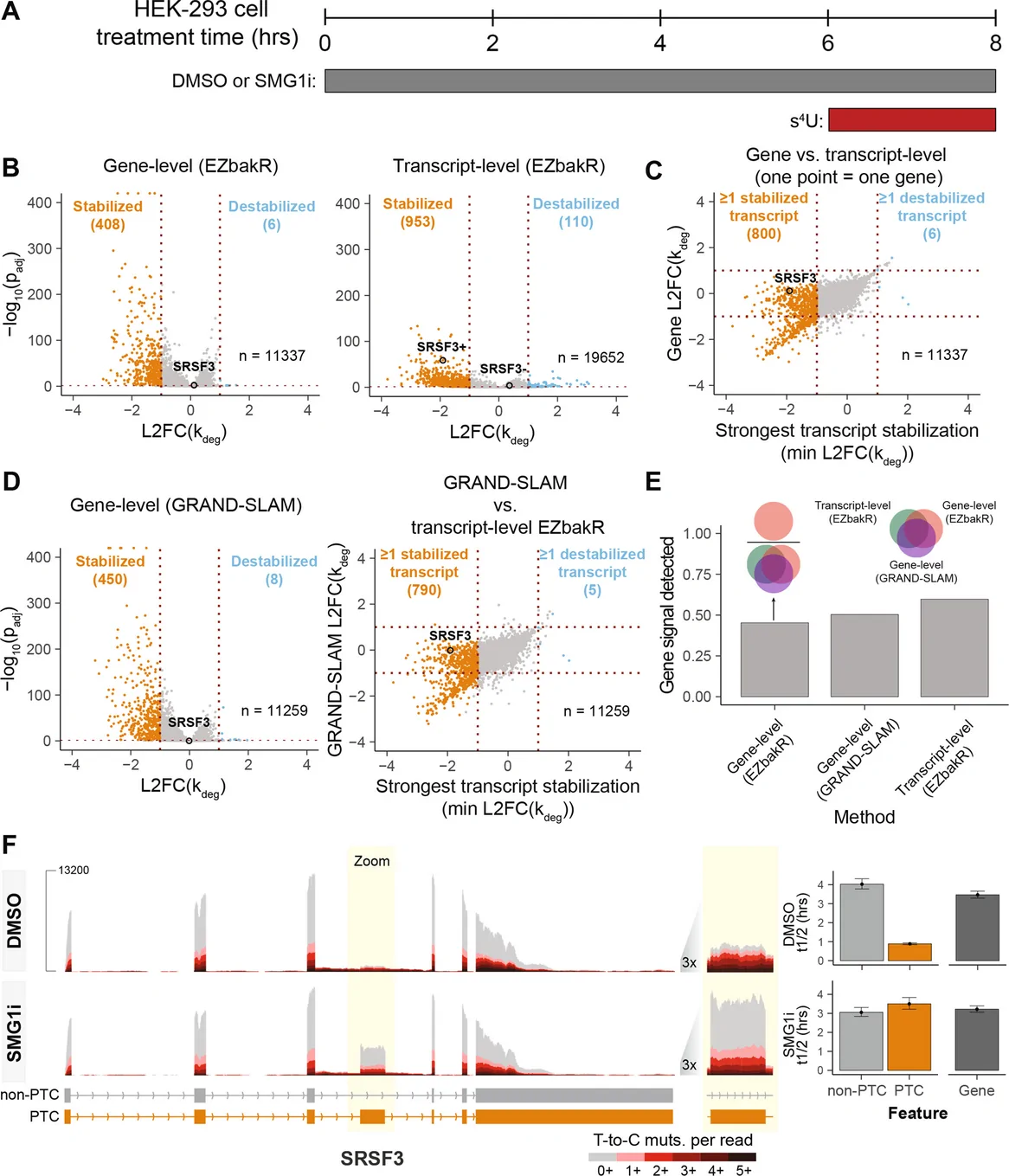
Isoform-level NR-seq analyses accurately identify targets of NMD. **A** Schematic for TimeLapse-seq experiment designed to identify targets of NMD. HEK-293 cells were treated with either a SMG1 inhibitor (SMG1i) or DMSO for 8 h. Cells were fed with s^4^U for the last 2 h of the treatment. **B** Gene- and isoform-level analysis volcano plots. L2FC(k_deg_) is log_2_(SMG1i k_deg_/DMSO k_deg_), i.e., the log_2_-fold change in the degradation rate constant estimate. Cutoff for significance is FDR < 0.01 and |L2FC(k_deg_)|> 1. For the gene-level analysis, only genes with at least 10 reads in all samples were included. For the isoform-level analyses, only isoforms with a TPM > 1 in all samples, as well as estimated read counts of 10 in all samples, were included. SRSF3 + refers to the PTC-containing isoform from the *SRSF3* gene, while SRSF3- is the non-PTC isoform. **C** Direct comparison of gene- and isoform-level analyses. For each gene, the minimum isoform L2FC(k_deg_) (i.e., strongest stabilization) was compared to the gene-level L2FC(k_deg_). **D** Same as (**B**) left and (**C**), but with gene-level analyses using GRAND-SLAM (multiple-test adjusted *p*-values calculated using EZbakR). **E** Comparison of the fraction of stabilization events identified by each analysis. A list of genes identified by any of the gene-level analyses or that produced at least one stabilized isoform was compiled. The fraction of these genes identified in each of the three analysis strategies tested was then calculated (y-axis). Venn diagrams schematize this scoring metric. Each circle represents the list of genes identified in each analysis. Bar plots represent the fraction of genes in one list present in the union of all lists, as depicted for the gene-level EZbakR analysis above the relevant bar. **F** Example sequencing tracks of an NMD target identified by the isoform-level analysis but missed by the gene-level analysis (*SRSF3*). Tracks are colored by mutational content. Bar plots to the right of the tracks depict the estimated isoform-level and gene-level half-lives (t_1/2_), in hours

The NMD pathway targets transcripts with specific features for RNA decay. Genes often produce a mixture of NMD-sensitive and NMD-insensitive transcripts, generated through alternative splicing, polyadenylation, and other processes, suggesting that understanding NMD mechanisms and roles would benefit from isoform-level quantification of RNA synthesis and degradation [25]. We therefore applied EZbakR’s isoform-level analyses to the task of identifying targets of NMD. To do so, we combined NR-seq with acute NMD inhibition and used EZbakR to identify significantly stabilized transcript isoforms (Fig. 2A and Additional file 2: Table S1). We chose to inhibit NMD with a specific inhibitor of the SMG1 kinase (SMG1i), which prevents the phosphorylation of the key NMD factor UPF1 and inhibits NMD [26]. Because this approach allows for analysis of RNA decay following a short period of NMD inhibition, we reasoned that it would primarily capture direct effects of blocking the pathway, a prediction supported by our initial gene-level analyses, which revealed 408 genes with significant RNA stabilization but only 6 with significant destabilization (Fig. 2B, left).

To evaluate the benefits of EZbakR’s isoform-level analysis, we compared it to a standard gene-level analysis approach. We hypothesized that the isoform-level analysis would reveal NMD targets missed by gene-level analysis due to minor-isoform stabilization being obscured by data from unaffected isoforms. Both the isoform-level and gene-level analyses identified many stabilization events with high confidence (Fig. 2B and Additional file 2: Table S2). Despite this, we noticed that several genes with NMD-sensitive transcripts identified in the isoform-level analysis were missed by the gene-level analysis. This included *SRSF3*, which produces a well-established NMD-target isoform [27, 28] and will serve as a reference point throughout. Comparing the strongest isoform-level stabilization at each gene to the gene-level stabilization, we confirmed our hypothesis that while there was generally good agreement, a large number of gene-level effects were significantly smaller than the strongest isoform-level effect (middle-left section of Fig. 2C). The same trend is observed when comparing the isoform-level EZbakR analysis to a gene-level analysis using a different NR-seq analysis tool, GRAND-SLAM (Fig. 2D) [19]. To further explore this observation, we defined a “stabilized gene set” comprising genes identified as producing significantly stabilized RNA by the EZbakR-suite or GRAND-SLAM gene-level analyses or as producing at least one isoform identified as stabilized by the EZbakR-suite transcript-level analysis. Then from this comprehensive set of stabilization hits, we determined what fraction of these events were detected by each individual analysis (Fig. 2E). This metric revealed that the isoform-level analysis was able to successfully identify more stabilization events than any of the gene-level analyses. The enhanced performance of the isoform-level analysis likely reflects NMD-sensitive isoforms comprising a small fraction of their gene’s total isoform pool, even after NMD inhibition (Fig. 2F). Thus, EZbakR’s isoform-level analyses are able to capture NMD targets missed by a gene-level NR-seq analysis.

### NR-seq analysis uncovers signal invisible to RNA-seq

To assess the value of having isoform kinetic parameter estimates in addition to abundance estimates, we investigated the relationship between differential expression and differential stabilization in response to SMG1 inhibition. We found a strong correlation between changes in isoform abundance and isoform degradation rate constants (Additional file 1: Fig. S2B, left). In particular, SMG1 inhibition resulted in a significant increase in both the abundance and stability of 554 isoforms. Despite this, there were 92 isoforms for which abundance significantly increased but there was little to no increase in stability (Additional file 1: Fig. S2B, right). These could represent indirect transcriptional effects of SMG1 inhibition that would have confounded a standard RNA-seq analysis. In addition, 151 isoforms appeared to be significantly stabilized while showing no increase in abundance. Some of these isoforms may represent instances of transcriptional repression in response to NMD inhibition that violates the steady-state assumption made by EZbakR, a challenge that can be curtailed with a slight modification to the NR-seq experimental design used here [29]. Alternatively, these may be instances of homeostatic mechanisms that couple transcription and degradation to maintain RNA levels [30–32]. This analysis shows how NR-seq can improve identification of NMD targets.

NR-seq and EZbakR also allowed us to assess the extent to which the turnover kinetics of an isoform contribute to its abundance. We found isoform degradation rate constants to be strongly negatively correlated with both isoform abundance (Additional file 1: Fig. S2C, left) and isoform fraction (the fraction of transcripts from a gene contributed by a specific isoform; Additional file 1: Fig. S2C, middle). To investigate this further, we quantified the coefficient of determination (R^2^; proportion of variance in data explained by a linear fit) for each of these relationships (Additional file 1: Fig. S2C, right). Isoform degradation rate constant differences explain around 38% of the isoform abundance variance, meaning that isoform turnover kinetics are a significant contributor to isoform expression levels. In addition, isoform degradation rate constant differences account for around 18% of the isoform fraction variance. This suggests not only that differences in the stability of isoforms from different genes are major determinants of the relative abundances of those isoforms, but also that differences in stabilities of isoforms from the same gene (and not just alternative splicing preferences) play a considerable role in establishing alternative isoform abundance. These analyses reveal that isoform turnover regulation is an important determinant of isoform expression levels.

### AnnotationCleaner: a pipeline for building custom annotations

Because the analysis in Fig. 2 used the RefSeq hg38 annotation, we suspected that some unannotated NMD targets were missed. This concern is consistent with prior work showing that many NMD targets are absent from standard references [33–36]. In addition, recent work and our own observations indicated that discrepancies between references and the HEK-293 transcriptome were leading to inaccuracies in the isoform-level analyses [37, 38] (Additional file 1: Figs. S3 and S4A). We therefore sought ways to build and stringently filter custom annotations.

**Fig. 3:**
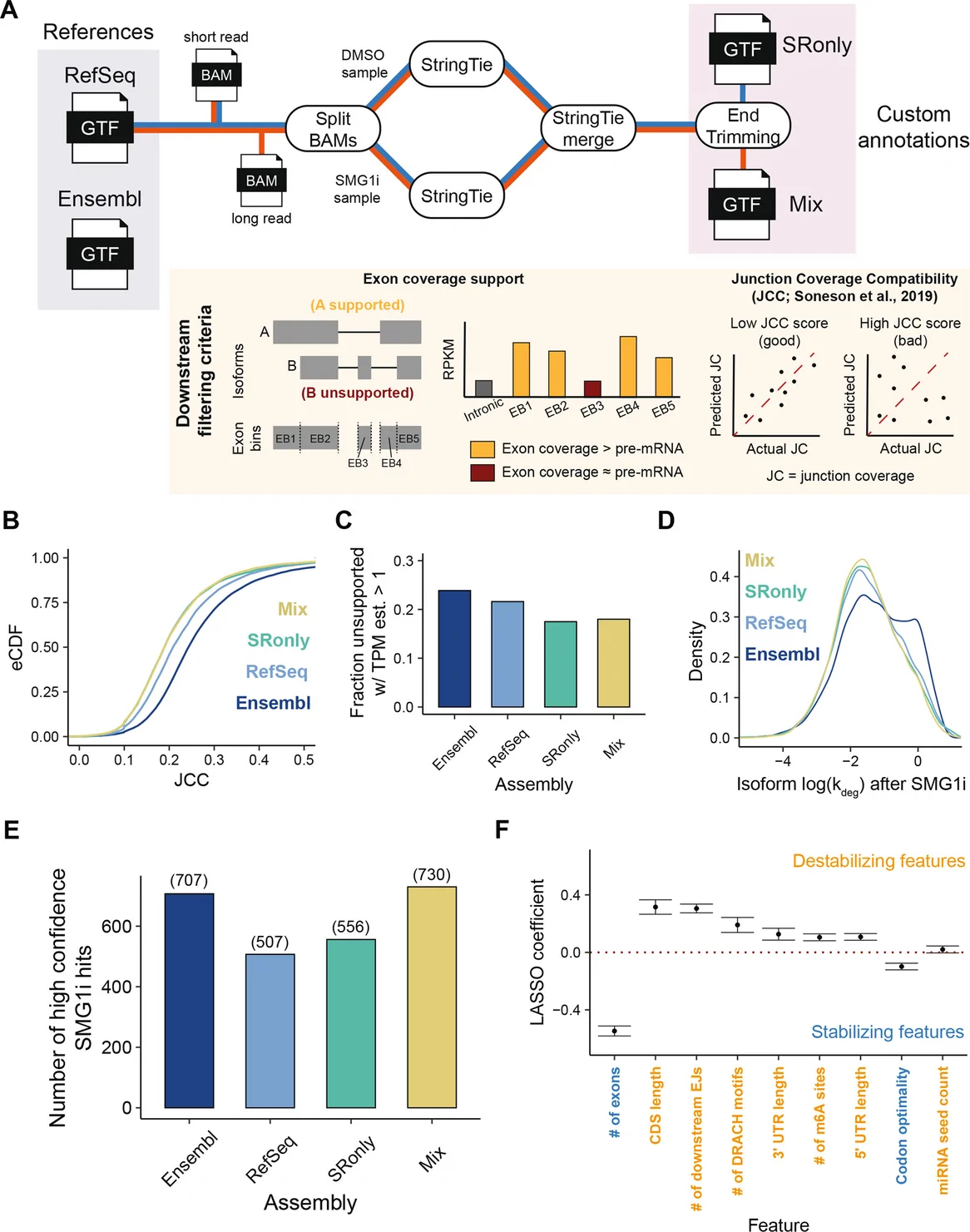
Annotation assembly and trimming improves isoform-level analyses. **A** Schematic of the AnnotationCleaner pipeline and downstream filtering criteria. Four annotations (2 references and 2 assemblies) were tested: Mix (StringTie using short and long-read data), SRonly (StringTie using only short-read data), RefSeq (hg38), and Ensembl (GRCh38 version 113). **B** Empirical cumulative distribution function (eCDF) plot comparing the JCC score distributions for all four annotations tested. Higher JCC scores represent worse isoform abundance vs. junction coverage discrepancy. **C** Comparison of fraction of isoforms deemed “unsupported” by AnnotationCleaner that have an RSEM estimated TPM > 1 in all samples. Unsupported isoforms are those with at least one exon lacking greater than pre-mRNA levels of coverage in all samples. **D** Comparison of the distribution of degradation rate constant estimates in SMG1i-treated cells, for all four annotations tested. **E** Comparison of the total number of high-confidence SMG1i sensitive isoforms for each annotation. High-confidence SMG1i sensitive isoforms are those with a L2FC(k_deg_) < −1, FDR < 0.01, and that pass both the unsupported isoform and JCC filtering (latter means expressed from a gene with JCC < 0.4). **F** LASSO regression model fit using the top performing annotation (Mix). Features are colored by whether increases in the feature value are associated with increases (destabilizing) or decreases (stabilizing) in k_deg_. LASSO coefficients indicate the relative contribution of each feature to the model-predicted DMSO-treated k_deg_, holding all other features constant

Towards that end, we developed AnnotationCleaner, a pipeline to refine references with short and long-read RNA-seq data. AnnotationCleaner combines reference-guided transcriptome assembly using the extensively validated tool StringTie with custom scripting to augment StringTie’s trimming of UTRs and flag poorly supported isoforms that should be filtered out in downstream analyses [39, 40] (Fig. 3A). Filtering was performed in two ways. First, we developed a new metric that identifies isoforms with low coverage (i.e., similar to pre-mRNA levels) of one or more exons and thus flags isoforms that do not have ample read coverage support in our RNA-seq data. Second, we used the previously described junction coverage compatibility (JCC) score–a metric used to evaluate the reliability of transcript-level abundance estimates and accuracy of transcript annotations–(Fig. 3A; see Methods for details) to flag genes with read coverage distributions deviating from those predicted by the estimated relative isoform abundances [41, 42].

### AnnotationCleaner improves isoform-level analyses

We used AnnotationCleaner to build two annotations, one using only short-read RNA-seq data (SRonly) and the other using both short and long-read RNA-seq data (Mix), which has been shown to improve ab initio assembly [40]. In both cases, we used the RefSeq annotation to guide this process and RNA-seq data from both DMSO- and SMG1i-treated cells (Fig. 3A). We then analyzed our NR-seq data with these two annotations and references from RefSeq and Ensembl [43, 44]. Comparison of JCC score distributions revealed that AnnotationCleaner lowered average JCC scores, indicating improved agreement between observed junction-level read coverage and coverage predicted from the isoform abundance estimates (Fig. 3B).

We then assessed the fraction of isoforms in each annotation with an RSEM-estimated TPM greater than one that had at least one exon with read coverage at or below that of pre-mRNA produced from the same gene. We term these “unsupported isoforms” and suspect that many of these are unexpressed isoforms erroneously assigned read coverage due to annotation discrepancies. Among the references, Ensembl had a higher fraction of such isoforms, which we believe is due to its higher gene model complexity and total isoform count (Fig. 3C and Additional file 1: Fig. S3). Many spurious isoforms contained exons overlapping highly transcribed intronic sequences, which risk producing rate constant estimates highly biased by the high fraction of labeled reads in these regions (Additional file 1: Fig. S3). Both of the custom annotations had significantly lower rates of unsupported isoforms, suggesting that StringTie’s filtering and refinement of the annotation improved isoform quantification (Fig. 3C).

To test whether annotation choice affects degradation rate constant inference, we assessed isoform turnover kinetics upon SMG1i treatment (Fig. 3D). Mix, SRonly, and RefSeq annotations yielded similar degradation rate constant distributions, but the Ensembl analysis differed substantially. Specifically, the Ensembl analysis was plagued by a large number of apparently highly unstable isoforms that likely represent pre-mRNA (Fig. 3D and Additional file 1: Figs. S3 and S4B, left and middle panels). These spurious annotation-dependent results further support the efficacy of StringTie-based annotation filtering prior to isoform-level analyses of RNA-seq and NR-seq data. While the more conservative RefSeq annotation yielded better metrics than Ensembl, it also had far fewer high confidence NMD targets than either of the AnnotationCleaner annotations (Fig. 3E). This highlights the benefits of expanding a more conservative reference with ab initio assembly. In addition, despite Ensembl having far more isoforms, it still yielded fewer high confidence NMD targets than the Mix assembly (Fig. 3E). Finally, we note that while including long-read data considerably improves identification of high confidence NMD targets, SRonly also had improved metrics relative to either of the references (Fig. 3C, D). Thus, even if long-read data is not readily available, short-read ab initio assembly can still significantly improve isoform quantification accuracy. All these observations led us to conclude that AnnotationCleaner improves EZbakR’s isoform-level NR-seq analyses.

Finally, we sought to further validate the isoform kinetic parameter estimates obtained using the top-performing AnnotationCleaner assembly (Mix). Towards that end, we evaluated whether our analyses aligned with other studies with respect to which features affect isoform stability. To associate transcript features with stability in DMSO-treated cells, we fit a LASSO regression model using our isoform degradation rate constant estimates (Fig. 3F). We considered the impact of a number of features previously identified to correlate with RNA stability (length, number of exons, m6A sites, etc.). We found the strongest covariate with stability to be the number of exons, with more exons being associated with greater stability. This supports recent work suggesting that EJCs inhibit m6A deposition and stabilize isoforms [45–48]. In addition, we corroborated previous observations that overall transcript length and m6A levels negatively correlate with stability, and that codon optimality positively correlates with isoform stability [49]. We were also able to orthogonally validate these conclusions using previously published RNA-seq transcription inhibition data, suggesting that none of these correlations arise as an artifact of metabolic labeling (Additional file 1: Fig. S4C, D) [50]. Together, these analyses support the conclusion that isoform-level NR-seq analyses using our custom annotation accurately quantify isoform turnover kinetics.

### NMD is a major determinant of isoform decay rate variability within genes

**Fig. 4:**
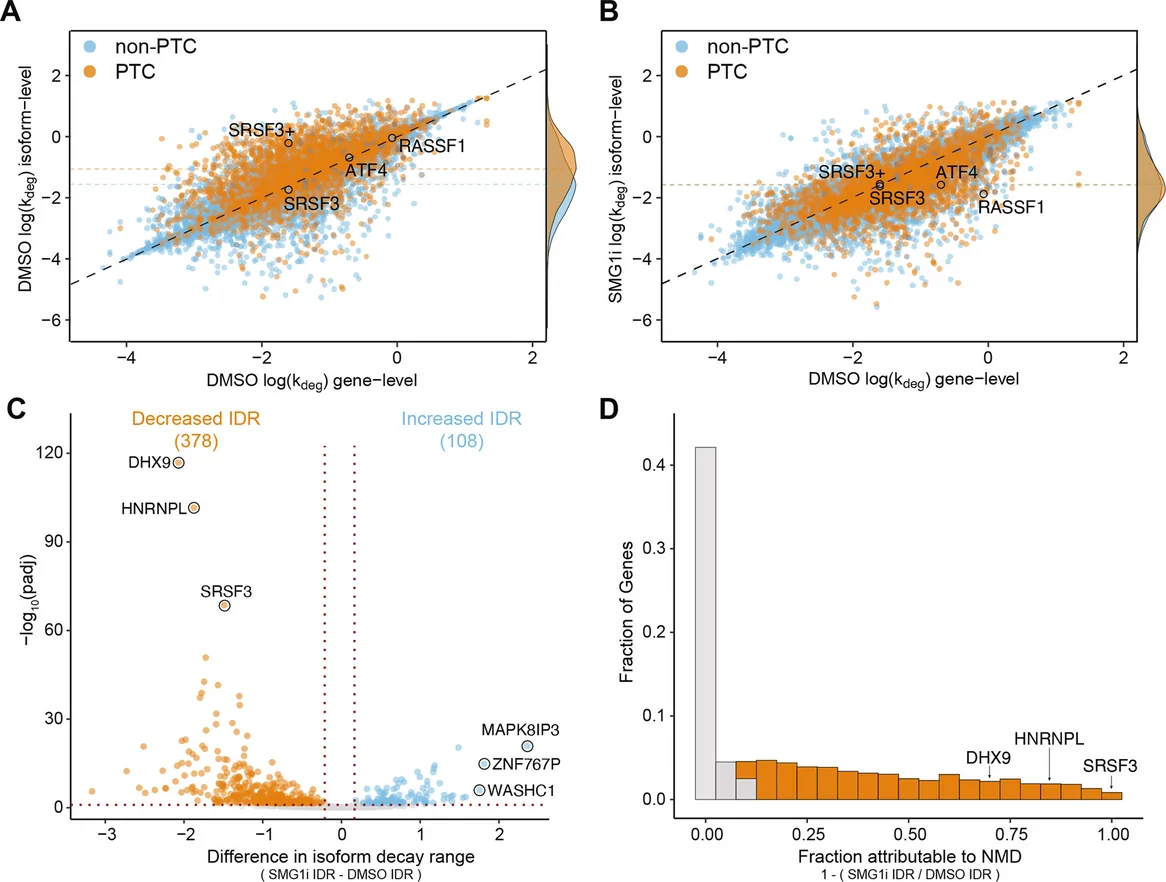
NMD plays a crucial role in driving variation in isoform stability within genes. **A**, **B** Scatter plots comparing isoform-level degradation rate constants (k_deg_) to gene-level degradation rate constants under DMSO (**A**) or SMG1i (**B**) treatment conditions. Isoforms are color-coded as non-PTC (blue) or PTC-containing (orange). The diagonal black line represents a linear relationship between gene-level and isoform-level k_deg_. Two *SRSF3* isoforms are highlighted: the non-PTC isoform (SRSF3 −) and the PTC-containing isoform (SRSF3 +), along with representative examples of NMD-sensitive transcripts harboring a uORF (*ATF4*) or a long 3′UTR (*RASSF1*). Density plots on right y-axes show the distribution of PTC and non-PTC isoforms. Horizontal orange and blue lines indicate the median value for PTC and non-PTC isoforms. **C** Volcano plot showing the difference in isoform decay range (IDR; SMG1i—DMSO) on the x-axis and -log_10_(padj) significance on the y-axis. Points represent multi-isoform genes (*n* = 3,966), with significant differences defined by padj < 0.05 and IDR > 0.1 (blue) or IDR < −0.1 (orange). Highlighted genes are discussed in the text. **D** Histogram depicting the distribution of isoform stability differences attributable to NMD. Orange bars indicate cases where more than 10% of the stability difference is attributed to NMD. *SRSF3* is discussed in the text

NMD plays a key role in regulating isoform stability by selectively targeting PTC-containing and select non-PTC transcripts, where PTC transcripts are defined as those in which the termination codon (TC) occurs more than 50 nt upstream of the last exon junction [6]. However, the isoform-level kinetics of NMD have not been systematically measured, and how NMD fits into the overall kinetic landscape of mRNA degradation is unknown. To investigate these questions, we first compared the turnover kinetics of PTC and non-PTC isoforms under DMSO treatment (Fig. 4A). Isoform-level degradation rate constants generally correlated with gene-level averages, but a subset of both PTC and non-PTC transcripts exhibited lower stability than their gene-level averages (higher isoform-level DMSO log(k_deg_) values), consistent with the characteristic instability of NMD substrates.

The stability profiles of PTC and non-PTC isoforms were distinct (Fig. 4A, median lines), with PTC isoforms often exhibiting much lower stabilities, as exemplified by *SRSF3*. Comparison of SMG1i TimeLapse-seq with published UPF1-AID SLAM-seq [51] revealed strong concordance for both PTC-containing and non-PTC transcripts, reinforcing that the effects observed upon SMG1 inhibition are predominantly due to NMD inhibition (Additional file 1: Fig. S5 and Additional file 2: Table S3). This correlation was evident for PTC-containing transcripts after 12 or 24 h of UPF1 depletion. These findings further highlight that gene-level averaging of stability measurements obscures isoform-specific variation in decay rate measurements across the transcriptome and that our analyses capture isoforms with distinct stabilities.

We investigated the extent to which NMD explained instances of isoform-to-isoform stability variance within the same gene (Fig. 4B). Following SMG1 inhibition, many isoforms were significantly stabilized, with their decay rate constants equal to or less than their gene-level averages (shift toward or below the diagonal). SMG1i treatment globally increased the stability of PTC isoforms, bringing their distribution of stabilities to match that of non-PTC isoforms (Fig. 4B, right; compare PTC and non-PTC median lines). This indicates complete inhibition of NMD upon SMG1i treatment for many mRNAs.

Given the prevalence of SMG1i-sensitive isoforms, we sought to directly quantify NMD’s transcriptome-wide contribution to RNA decay. To do this, we calculated an Isoform Decay Range (IDR) for each gene (see Methods for details), defined as the difference in isoform stability (i.e., between the most and least stable isoforms) within a gene. We then compared IDR changes between DMSO and SMG1i treatments (Fig. 4C and Additional file 2: Table S4). This analysis revealed that of all multi-isoform genes, 378 genes exhibited a significant decrease in IDR, whereas only 108 showed an increase. Decreased IDR observations were associated with reduced stability differences between PTC and non-PTC isoforms within the same gene (e.g., *SRSF3*, *DHX9*, *HNRNPL*). Notably, several of the largest IDR increases occurred in genes where multiple PTC isoforms displayed variable basal stabilities and responded to SMG1 inhibition to different extents (e.g., *MAPK8IP3*, *ZNF767P*, *WASHC1*). To further estimate the impact of NMD on gene-level isoform stability differences, we calculated the fraction of gene-level IDR attributable to NMD (Fig. 4D). This analysis revealed that 50.9% of genes with multiple well-expressed isoforms exhibited a decrease in IDR of 10% or more, with this difference in isoform stabilities attributable to SMG1i treatment. For instance, in *SRSF3*, nearly all stability differences between the PTC (*SRSF3* +) and non-PTC (*SRSF3 −*) isoforms were driven by NMD, with SMG1i treatment increasing the stability of the PTC isoform to match that of the non-PTC isoform (Fig. 4B, D). More broadly, this analysis indicates that NMD influences isoform stability differences in over half of all multi-isoform genes expressed in HEK-293 cells.

### Basal transcript instability constrains NMD

Leveraging our improved isoform-level measurements of NMD kinetics, we evaluated previously proposed rules for NMD. We separated transcripts into PTC and non-PTC isoforms and used linear regression to assess correlations between NMD efficiency and various parameters reported to influence NMD, including lengths of the coding sequence (CDS), TC-containing exon, longest internal exon, and 3′UTR. In doing so, we sought to determine whether the correlations observed in previous studies using RNA abundance data were evident using our measurements of turnover kinetics.

We found that PTC-containing isoforms with longer CDS lengths showed greater stabilization under SMG1i treatment compared to those with shorter CDS lengths, while non-PTC isoforms did not exhibit this correlation (Additional file 1: Fig. S6A). This supports previous findings that mRNAs with CDS lengths shorter than 100 nucleotides are less efficient NMD substrates [9], due to re-initiation at downstream start codons or other factors [52–54]. Also consistent with previous analyses, PTC-containing isoforms with more downstream exon-junctions (Additional file 1: Fig. S6B) and shorter distances between the termination codon and the nearest downstream exon-junction (Additional file 1: Fig. S6C) were more strongly stabilized by SMG1i [9, 55].

**Fig. 5:**
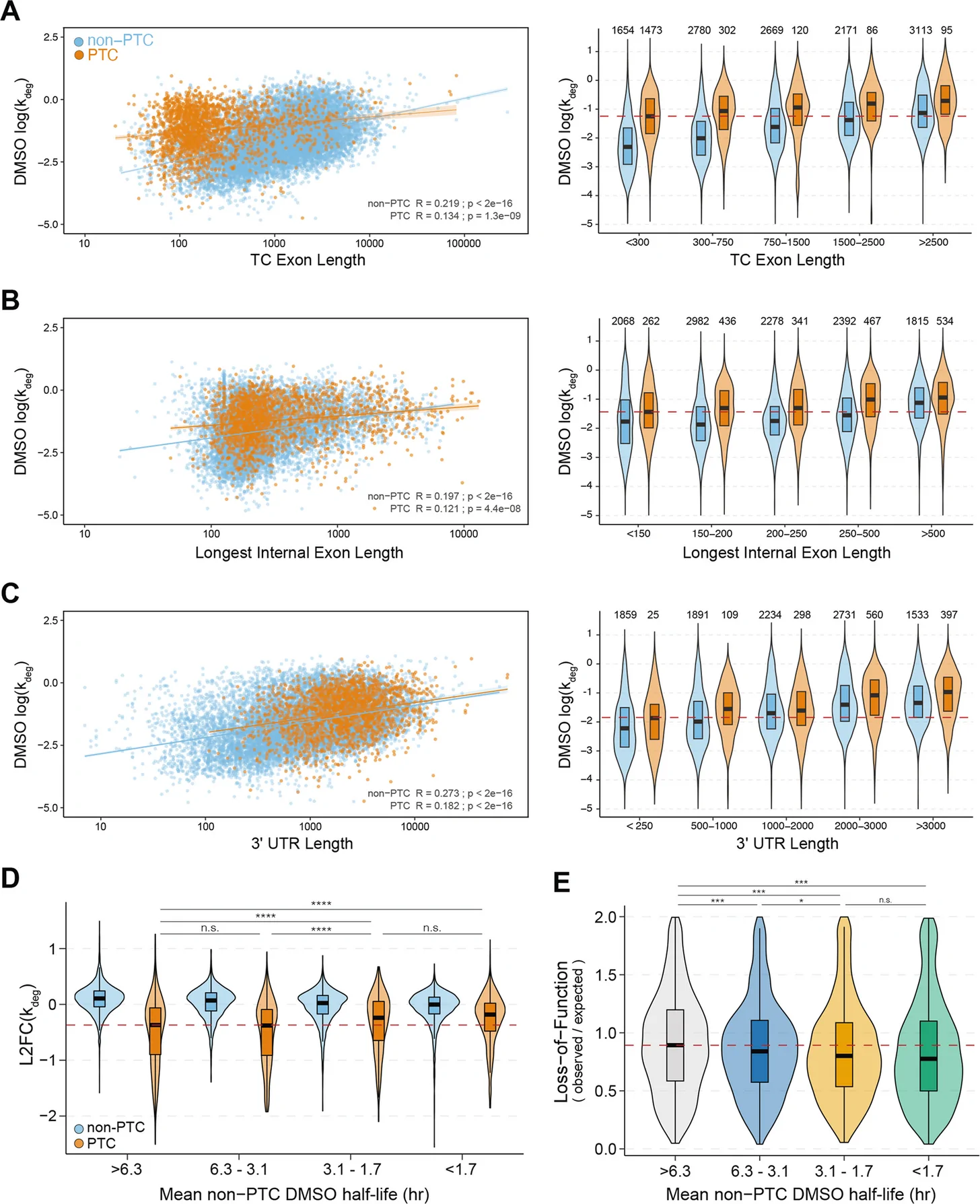
Intrinsic transcript instability limits NMD efficiency. **A**-**C** Left: Scatter plots depicting basal DMSO log(k_deg_) estimates as a function of **A** TC exon length, **B** longest internal exon length, and **C** 3′UTR length for non-PTC (blue) and PTC-containing (orange) isoforms. Linear regression fit lines with standard error shading are shown for both PTC and non-PTC data, along with corresponding correlation coefficients (R) and significance values (*p*-value) for each regression. Right: Violin plots displaying the distribution of basal DMSO log(k_deg_) estimates against length-wise binning of the same features **A**-**C** for non-PTC (blue) and PTC-containing (orange) isoforms. Dashed red lines denote the mean basal DMSO log(k_deg_) of the shortest feature length bin of PTC isoforms. **D** Violin plots showing the distribution of L2FC(k_deg_) values between SMG1i and DMSO treatments for non-PTC (blue) and PTC-containing (orange) isoforms. Isoforms are binned based on the mean DMSO half-life of non-PTC isoforms from the same gene. The horizontal red dashed line represents the lowest median L2FC(k_deg_) observed across all bins. **E** Violin plots displaying the distribution of loss-of-function (observed/expected) scores for non-PTC isoforms, binned according to the mean DMSO half-life of non-PTC isoforms from the same gene. Statistical significance of pairwise comparisons between bins is indicated above the plots (n.s. not significant, * *p* < 0.05, ** *p* < 0.01, *** *p* < 0.001)

We then examined the relationship between the length of TC-containing exons and changes in isoform stability upon NMD inhibition. Isoforms with PTCs in longer exons were associated with reduced sensitivity to SMG1i, which corroborates previous findings [9] (Additional file 1: Fig. S6D). Importantly, we also observed significantly lower basal stability of PTC isoforms with long TC-containing exons than those with short TC-containing exons (Fig. 5A, left). This result suggests that transcripts with long TC-containing exons are subject to efficient NMD-independent turnover, to the extent that PTC-free transcripts with TC exon lengths greater than 1,500 nucleotides are often as unstable as PTC transcripts (Fig. 5A, right). We further tested this idea by examining the correlation between basal transcript stability and the length of the longest internal exon (Fig. 5B, left), finding a correlation for both PTC and non-PTC transcripts. Additionally, non-PTC isoforms with internal exons longer than 500 nucleotides show reduced basal stability similar to that of PTC-containing isoforms (Fig. 5B, right, and Additional file 1: Fig. S6E). Together, our data suggest that the observed reduction in NMD susceptibility of transcripts with PTCs in long exons may be due to the destabilizing effects of long exons rather than ineffective NMD per se. The same principle can be applied to the related parameter of distance between the TC and the nearest downstream exon junction.

Lastly, we analyzed 3′UTR length, a common characteristic implicated in decay of downstream EJC-independent NMD substrates. In our analysis, we observed a slight inverse correlation between 3′UTR length and the response of PTC or non-PTC isoforms to SMG1i (Additional file 1: Fig. S6F). However, when comparing 3′UTR length with basal RNA stability, we found a much stronger correlation, where longer 3′UTRs were associated with reduced basal RNA stability (Fig. 5C, left). Both PTC-containing and non-PTC isoforms followed this trend, with non-PTC isoforms with 3′UTRs longer than 2,000 nucleotides showing destabilization comparable to most PTC isoforms (Fig. 5C, right). These results suggest that isoforms with longer exons (TC-containing and internal) and 3′UTRs are prone to decay by other pathways, reducing their apparent sensitivity to NMD.

To globally evaluate how basal stability constrains NMD, we took advantage of the ability to measure the decay rate constants of PTC and non-PTC isoforms produced from the same gene. We binned genes based on the mean DMSO half-lives of their non-PTC isoforms and compared decay rate constant changes between SMG1i and DMSO treatments for both PTC and non-PTC isoforms (Fig. 5D). PTC-containing isoforms showed progressively less responsiveness to SMG1i as the basal stability of non-PTC isoforms from the same gene decreased (Fig. 5D). One example of basal instability influencing NMD sensitivity is *HMGXB4*. In this gene, the inclusion of a poison exon (i.e. an exon that encodes a PTC when included in mature mRNA), along with an upstream exon, creates an open reading frame that introduces a stop codon within the poison exon (Additional file 1: Fig. S6G). We found that all isoforms of *HMGXB4* are highly unstable (median isoform half-life of 53 min), with both non-PTC and PTC-containing isoforms exhibiting similar expression levels and higher decay rate estimates under DMSO treatment (Additional file 1: Fig. S6G, right; high ratio of s^4^U labeled to unlabeled reads). Furthermore, SMG1i treatment did not stabilize any of the *HMGXB4* isoforms, including the poison exon-containing isoform (Additional file 1: Fig. S6G, right). Taken together, these findings underscore the potential for competing RNA decay pathways to limit the capacity of NMD to regulate gene expression.

We next asked whether quality control may also be impaired by basal transcript instability, as previously suggested [9]. We used the gnomAD database to obtain gene-level loss of function observed/expected upper bound fraction (LOEUF) scores [56], a measure of a gene’s tolerance to inactivating mutations. We binned each gene by its mean non-PTC isoform DMSO half-life (Fig. 5E) and found decreased LOEUF scores among genes with shorter mean non-PTC isoform DMSO half-lives. The finding that genes with lower basal stability tend to be less tolerant to loss-of-function mutations is predicted by a model in which NMD is unable to effectively reduce the abundance and translation of PTC-containing transcripts from genes with low basal transcript stability.

### NMD efficiently targets a subset of non-PTC isoforms, including a novel class of PTC-free AS-NMD substrates

Current models suggest that decay of PTC-containing transcripts is inherently more efficient than decay of non-PTC NMD targets, due to decay stimulation by the EJC. By utilizing degradation rate constant measurements, rather than solely relying on RNA abundance changes, we could better evaluate the differences in stabilities between PTC and non-PTC NMD substrates. To make this comparison, we curated two sets of previously validated PTC-dependent and -independent NMD substrates. The first set consisted of transcripts containing previously identified conserved poison exons [57]. The second set included genes encoding NMD factors, most of which are autoregulated by the pathway, along with the previously characterized NMD substrate *IRE1* (*ERN1*). Several of these genes have 3′UTRs that have been shown in reporter assays to be sufficient to trigger decay in the absence of a PTC [58–61].

To identify additional high-confidence, SMG6-dependent NMD targets transcriptome-wide, we performed Akron5-seq [62], which quantitatively captures RNA 5′ ends at single-nucleotide resolution. To stabilize decay intermediates, the major 5′−3′ exonuclease XRN1 was depleted alone or in combination with SMG6. Our analysis revealed that SMG6-dependent peaks were enriched at both PTCs and regulated non-PTC termination codons and that decapping increased upon SMG6 depletion (Additional file 1: Fig. S7A and Additional file 2: Table S5), consistent with previous SMG6-cleavage mapping studies [63–65]. We compared changes in peak abundance between XRN1-depleted and XRN1/SMG6 co-depleted conditions with changes in isoform k_deg_ following SMG1 inhibition (Additional file 1: Fig. S7B), which revealed that a subset of non-PTC isoforms stabilized by SMG1 inhibition also exhibited detectable SMG6-dependent cleavage near annotated termination codons. We also analyzed a published dataset of SMG6 cleavage sites to further corroborate our findings [63]. This analysis enabled us to find a stringently filtered set of non-PTC isoforms that depend on SMG6 cleavage near termination codons, adhere to the 50-nucleotide rule, and exhibit sensitivity to SMG1 inhibition (Additional file 1: Fig. S7C-F; see Methods for details).

**Fig. 6:**
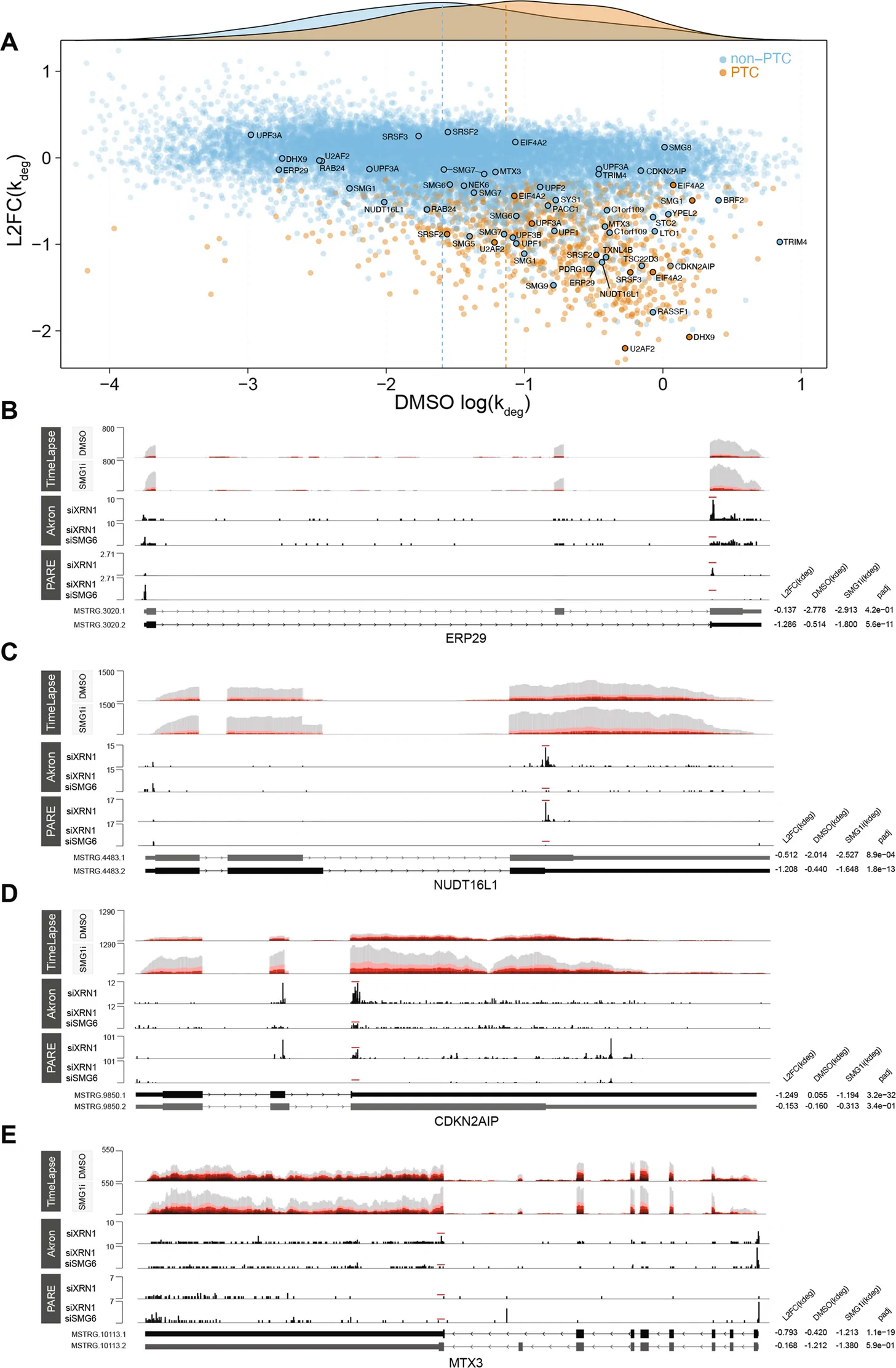
NMD efficiently regulates a subset of EJC-independent substrates. **A** Scatter plot comparing the L2FC(k_deg_) values between SMG1i and DMSO treatments (y-axis) to the basal DMSO log(k_deg_) estimates (x-axis) for non-PTC (blue) and PTC-containing (orange) isoforms. *Top*: Density plot displaying the distribution of DMSO log(k_deg_) estimates for PTC and non-PTC isoforms, with vertical lines indicating the median of each group's distribution. Genes highlighted in the figure are discussed further in the text and in Additional file 1: Fig. S7. **B**-**E** Top: TimeLapse-seq tracks for *ERP29* (**B**), *NUDT16L1* (**C**), *CDKN2AIP* (**D**), and *MTX3* (**E**) under DMSO (upper tracks) and SMG1i (lower tracks) treatment conditions. The degree of s^4^U labeling is indicated by increasing darkness of red, corresponding to the number of T-to-C mutations per read, with unlabeled reads shown in gray. Bottom: Akron5-seq (this study) and PARE-seq [63] tracks from *XRN1* and *XRN1* + *SMG6* co-depletion experiments, marking SMG6-dependent cleavage sites in Akron5-seq by red lines above the sequence tracks. The isoform from each gene is schematized below, with the ORFs represented by thicker lines. SMG1i-sensitive isoforms are colored black, while SMG1i-insensitive isoforms are gray. Corresponding decay rate measurements for these isoforms are displayed to the right

Next, we compared the basal stabilities and changes in decay rate constants upon SMG1i treatment for all predicted PTC and non-PTC isoforms (Fig. 6A and Additional file 2: Table S2). Globally, PTC-containing transcripts were generally more unstable and more strongly stabilized by SMG1i than non-PTC isoforms. Overlaying the aforementioned set of PTC and non-PTC decay substrates revealed that the set of poison exon-containing isoforms was, as expected, on average highly unstable under DMSO conditions and strongly stabilized by SMG1i. Notably, a subset of non-PTC NMD isoforms, including from the *TSC22D3*, *PDRG1*, *NUDT16L1*, *CDKN2AIP*, and *RASSF1* genes, were at least as unstable as validated PTC-containing isoforms and were similarly strongly stabilized by SMG1i (Fig. 6A) and UPF1 depletion in an independent dataset [51] (Additional file 2: Table S3). These findings suggest that NMD just as efficiently targets certain non-PTC substrates as it does PTC isoforms.

To orthogonally validate our findings, we assessed transcript stability by exclusively analyzing reads mapping to splice junctions (Additional file 2: Table S6), an approach that is less dependent on annotation quality and avoids potential rate constant biases from high turnover pre-mRNA. For this, we leveraged the ability of the EZbakR-suite to flexibly analyze reads mapping to various transcript features, including reads that span exon-exon splice junctions. Junction-level and isoform-level measurements revealed highly similar patterns of PTC and non-PTC transcript degradation by NMD, further supporting the accuracy of our isoform-level stability estimates (Additional file 1: Fig. S7G-J).

Isoform- and junction-level analyses consistently indicated that mRNAs with conserved poison exons have a broad range of half-lives in unperturbed cells, spanning from 45 min to over two hours. Correspondingly, the curated set of PTC isoforms also responded variably to SMG1i treatment (Additional file 1: Fig. S7G-J). These findings reinforce the conclusion that there are substantial differences in NMD efficiency even among high-confidence PTC transcripts. Junction-level analyses also supported the existence of highly efficient NMD of certain non-PTC transcripts, as nearly all curated non-PTC target mRNAs exhibited basal stabilities and responses to SMG1i within the interquartile ranges of the curated set of PTC mRNAs in both isoform- and junction-level analyses.

These analyses also revealed unexpected examples of genes for which differential termination codon positioning in terminal exons drives PTC-free alternative splicing-linked NMD (AS-NMD) (Fig. 6B-E). *ERP29*, *NUDT16L1*, *CDKN2AIP*, and *MTX3* each are alternatively spliced to express two isoforms, one SMG1i sensitive and one insensitive (Additional file 1: Fig. S7H). NMD-sensitive isoforms of these genes contain shortened open reading frames and extended 3′UTRs relative to the NMD-insensitive isoforms, creating TCs that are efficiently recognized by NMD despite adhering to the 50-nucleotide rule. We restricted our identification of PTC-free AS-NMD genes to those for which SMG6 cleavage sites unambiguously mapped to the upstream stop codon predicted to be used in the stabilized transcript isoform (Fig. 6B-E, Akron and PARE tracks). To further verify these results, we analyzed published data from *SMG7* knockout cells with or without additional *SMG5* knockdown, finding that each of the PTC-free AS-NMD targets identified are sensitive to depletion of core NMD factors [66] (Additional file 1: Fig. S7K-P and Additional file 2: Table S7). This demonstrates how cells can achieve robust isoform-specific regulation by coupling alternative splicing to efficient NMD in a downstream exon junction-independent manner.

### Machine learning identifies covariates of isoform-specific NMD kinetics

To identify covariates of PTC-dependent and independent NMD, we identified a subset of transcripts that were either high-confidence instances of SMG1i sensitivity or insensitivity and then fit a LASSO binary classification model to either the set of all PTC or non-PTC containing isoforms of these subsets. The model included a mix of established factors from the literature and new variables that we hypothesized could influence NMD (Additional file 2: Table S8). We evaluated model performance using held-out test data from one hundred bootstrapped samples (see Methods). Receiver operating characteristic (ROC) curves (the true positive rate versus the false positive rate across classification thresholds) and precision–recall curves (the precision (1—FDR) versus the true positive rate across classification thresholds) were used to assess how well each model distinguished NMD-sensitive from insensitive transcripts (Additional file 1: Fig. S8A, B). The PTC model performed extremely well (AUROC = 0.982; AUPRC = 0.986), indicating very high accuracy. The non-PTC model also performed well, though with lower overall accuracy (AUROC = 0.855; AUPRC = 0.841), consistent with the observed heterogeneity among non-PTC substrates.

**Fig. 7:**
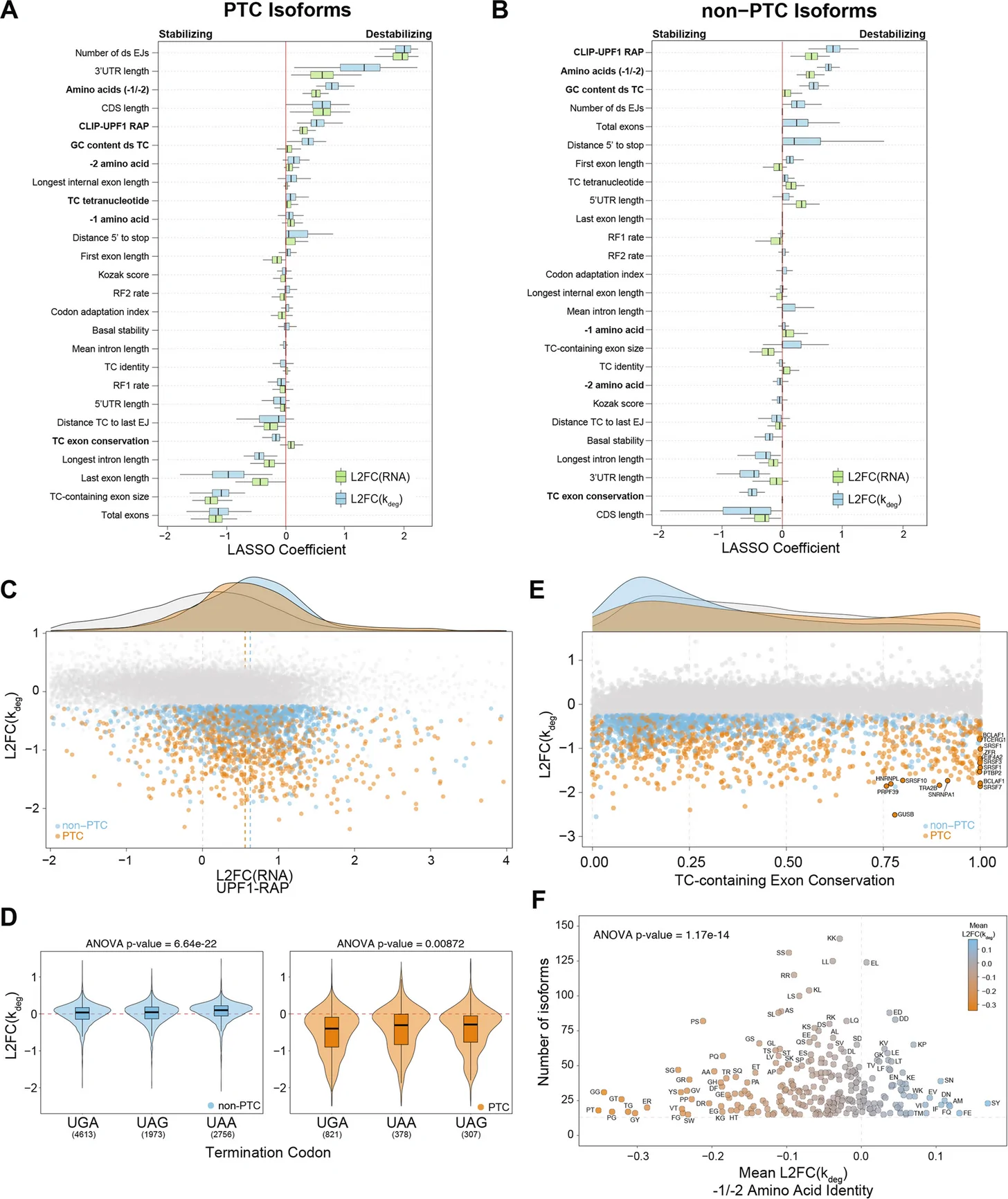
Machine learning reveals key features of PTC and non-PTC isoform-specific NMD kinetics. **A**, **B** Box plots showing the estimated LASSO regression coefficients (100 bootstrapped resamplings) for L2FC(k_deg_) (blue) and L2FC(RNA) in PTC (**A**) and non-PTC (**B**) isoforms for tested features (left). LASSO coefficients are interpretable as the extent to which the model predicted probability of SMG1i sensitivity changes when that feature is changed, holding all other features constant. **C** Scatter plot showing L2FC(k_deg_) between SMG1i and DMSO treatments versus L2FC(RNA) enrichment from CLIP-UPF1 RNA affinity purification (RAP). Non-PTC isoforms are represented in blue, PTC isoforms in orange, and non-responsive isoforms in gray. Vertical lines indicate the median of each group's distribution. Top: Density plot of each group with vertical lines indicating the median of each group's distribution. **D** Violin plots showing the distribution of L2FC(k_deg_) between SMG1i and DMSO treatments for different TC identities. Left plots (blue) are for non-PTC isoforms and right plots (orange) are for PTC isoforms. The horizontal dashed red line represents no change in the k_deg_ between conditions. The number of observations for each stop codon (TC) identity are shown below the plots. ANOVA *p*-value indicates statistical significance after assessing whether the variance between TC groups exceeds the variance within TC groups (i.e., whether there is strong evidence that different TC groups have different average L2FC(k_deg_) values). **E** Scatter plot of L2FC(k_deg_) versus TC-containing exon conservation scores. Non-PTC isoforms are shown in blue, PTC isoforms in orange, and non-responsive isoforms in gray. Top: Density plot for each group, with vertical lines indicating the median of each distribution. A subset of highly conserved TC-containing exon isoforms is labeled. **F** Scatter plot of mean L2FC(k_deg_) for each C-terminal amino acid pair versus the number of isoforms containing them. Amino acid pairs are colored by their mean L2FC(k_deg_), as indicated in the legend (*top right*). ANOVA *p*-value reflects statistical significance as described in (**D**)

As expected, the strongest predictor of PTC-containing isoform NMD efficiency was the presence of a downstream exon junction (Fig. 7A). The model also predicted other important factors previously linked to decay of PTC-containing RNAs [9, 11], including the number of downstream exon junctions, the distance between the PTC and the mRNA 5′ end, and 3′UTR length, validating our approach to transcript classification and machine learning. However, features recently proposed to be positively associated with NMD susceptibility due to pervasive mis-splicing, such as total exon number and intron length, did not significantly contribute to our model’s predictions [67]. CDS length had opposite correlations in PTC and non-PTC isoforms: longer CDS lengths were positively correlated with NMD sensitivity in PTC-containing isoforms, whereas longer CDS lengths correlated with lower NMD efficiency in non-PTC isoforms (Fig. 7A, B and Additional file 2: Table S8). This demonstrates that our transcript classification can effectively distinguish between correlates of PTC and non-PTC NMD substrates, capturing both shared and distinct regulatory features.

### UPF1 association correlates with decay of PTC and non-PTC transcripts

To further assess how various features relate to NMD susceptibility of PTC- and non-PTC-containing isoforms, we examined RNA–protein interactions using our previously published UPF1 RNA affinity purification and sequencing (RAP-seq) dataset [68, 69]. We observed a strong positive correlation between UPF1 recovery and the decay of both PTC- and non-PTC-containing transcripts (Fig. 7C). Notably, previously characterized PTC and non-PTC NMD substrates were enriched in UPF1 RAP-seq data (Additional file 1: Fig. S8C), supporting prior findings that UPF1 preferentially associates with NMD-susceptible mRNAs [70, 71].

### Nucleotide and amino acid sequences associated with efficient NMD

In addition to RNA–protein interactions, we identified multiple mRNA sequence-based features that influence NMD susceptibility. We observed a significant difference in GC content within the 200 nucleotides downstream of termination codons, which was strongly associated with stability changes but not RNA abundance changes (Fig. 7A, B). Non-responsive isoforms exhibited a median GC content of 44%, whereas responsive PTC and non-PTC isoforms had significantly higher GC contents at 50% and 54%, respectively (Additional file 1: Fig. S8D), consistent with previous findings on the role of 3′UTR GC content in modulating NMD efficiency [72, 73].

Termination codon identity was significantly associated with differential SMG1i sensitivity, with both PTC and non-PTC isoforms containing UGA stop codons exhibiting the strongest response to SMG1i treatment (Fig. 7D). Further, LASSO regression identified TC tetranucleotide context (TC identity plus the following + 4 nucleotide) as a stronger correlate of PTC and non-PTC NMD efficiency than TC identity alone. Among non-PTC targets, mRNAs with UGAC tetranucleotides were most strongly affected by SMG1i treatment. Our evidence of preferential decay of endogenous transcripts with UGA TCs, particularly those with a + 4 C nucleotide, corroborates the results of a recent massively parallel reporter assay of EJC-dependent and independent substrates [74] (Additional file 1: Fig. S8E). Notably, UGAC stop codons support less efficient termination than other tetranucleotide contexts, suggesting that the NMD machinery may be better able to recognize termination events with slow kinetics [75–78].

The identification of several sequence features associated with NMD susceptibility suggested that mRNA sequences may evolve to tune NMD efficiency. In support of this hypothesis, we found that sequence conservation of the exon containing the stop codon was strongly anti-correlated with RNA stability in both PTC and non-PTC isoforms, whereas RNA abundance showed little to no enrichment for exon conservation scores. We observed a bimodal distribution of SMG1i-sensitive PTC isoforms, consisting of a large population of poorly conserved exons and a notable subset of very highly conserved exons, many corresponding to known poison cassette exons in RNA-binding protein genes (e.g. SR protein genes, *PTBP2*, *EIF4A2*, and *BCLAF1*) (Fig. 7E). These findings reflect the diverse ways in which human cells use NMD to both maintain gene expression fidelity and enforce gene expression regulation.

Finally, the model also identified C-terminal dipeptide sequence context as a correlate of NMD susceptibility (Fig. 7A, B). Both PTC and non-PTC isoforms exhibited enrichment of specific residues at the minus one and minus two positions relative to the TC, with glycine, threonine, cysteine, and serine being enriched at the minus one position, while glycine, cysteine, and proline were enriched at the minus two position (Additional file 1: Fig. S8F, G). Notably, glycine and proline were particularly enriched in many of the dipeptide identities that were highly sensitive to SMG1i treatment (Fig. 7F). Transcripts encoding C-terminal glycine residues were most strongly stabilized by SMG1i (Additional file 1: Fig. S8F), consistent with the recent identification of terminal glycine residues as important modifiers of NMD efficiency [74]. These findings further suggest that additional aspects of amino acid sequence context may play a significant role in modulating transcript stability.

Taken together, these analyses highlight the power of kinetic measurements in resolving NMD covariates. Features such as stop codon exon conservation and downstream GC content, which were strong correlates in our kinetic data, were either weakly correlated or displayed opposite trends when analyzed using RNA abundance. These findings reinforce the importance of isoform-specific decay kinetics in understanding transcriptome-wide NMD regulation and reveal a complex interplay among RNA sequence features, exon conservation, and UPF1 association in shaping NMD efficiency across diverse transcript classes.

## Discussion

RNA abundance measurements have been instrumental in shaping our understanding of gene expression mechanisms. However, RNA abundance is an indirect readout and can therefore generate an incomplete or misleading picture of the contributions of RNA biogenesis and decay to gene expression regulation. In this work, we present the first strategy capable of inferring transcript isoform synthesis and degradation kinetics from short-read NR-seq of total RNA. We have implemented this strategy in our recently developed EZbakR-suite to facilitate its wide adoption among researchers. We envision that NR-seq and EZbakR will be powerful and broadly applicable tools to understand mechanisms and cellular functions of gene expression regulatory pathways.

Our findings underscore how the choice of annotation can significantly impact isoform-level investigations of RNA-seq data. We have developed AnnotationCleaner to increase representation of PTC isoforms that are often omitted from reference annotations while culling spurious transcript models. We find that it is particularly important to limit the contribution of isoforms that likely reflect unstable pre-mRNAs when estimating mature mRNA decay rate constants, an issue we find to be prevalent in Ensembl annotations. For most applications, we advocate for starting from a more conservative annotation and expanding it with tools like StringTie to generate custom references for each biological system of interest. In the Mix annotation used for this work, we have emphasized accuracy over completeness, but different experimental systems or research questions may require different approaches to annotation assembly and filtering.

While previous studies have primarily inferred NMD sensitivity from changes in steady-state RNA levels, our findings emphasize the need for direct kinetic measurements to fully capture the dynamics of NMD-mediated transcript turnover. Using EZbakR, we analyzed transcript synthesis and degradation kinetics at isoform and sub-isoform resolution, integrating NR-seq with acute NMD inhibition to reveal the kinetic effects of NMD. Our findings reveal that while PTC-containing isoforms are generally susceptible to NMD, conserved poison exon-containing isoforms exhibit substantial variability in decay rates. We find that increased numbers of downstream exon junctions and increased 3′UTR length are associated with greater likelihood of efficient decay of PTC-containing transcripts. Importantly, we also identify a subset of non-PTC isoforms that are as unstable as PTC-containing transcripts and are equally stabilized upon SMG1i treatment, challenging the assumption that non-PTC isoforms are necessarily less efficiently targeted by NMD. These include what are, to our knowledge, the first known examples of PTC-free AS-NMD in mammalian cells. Notably, the NMD-sensitive isoforms of three of the four PTC-free AS-NMD genes identified here contain C-terminal glycine residues, further indicative of selection for efficient decay [74]. These insights expand our understanding of NMD-based regulation and highlight the complexity of transcript features that dictate susceptibility to degradation.

The potential for basal transcript instability (i.e., the efficiency with which non-NMD decay pathways act on a transcript) to constrain NMD has been previously recognized [9]. However, EZbakR offers the important advantage of providing accurate measurements of the stability of PTC and non-PTC isoforms from the same gene. This approach identifies PTC-containing transcripts that are likely resistant to NMD due to competing turnover mechanisms and reveals mechanisms contributing to known NMD efficiency correlates. Notably, our data suggest that the apparent inefficiency of NMD of transcripts with long PTC-containing exons is largely attributable to basal instability. We find that mRNAs with long internal exons, irrespective of TC position, are often as unstable as NMD-sensitive PTC transcripts. In addition, our data provide a mechanistic explanation for recent observations that loss of function-intolerant genes are less sensitive to NMD [10, 56].

Putting NMD in the context of global transcript decay rate constants also sheds light on the regulation of mRNAs with long 3′UTRs. We find that transcripts with long 3′UTRs, like those with long internal exons, often exhibit decay rate constants similar to those of well-targeted PTC transcripts. These results are consistent with a recent report of NMD-independent decay of long 3′UTRs [79]. Thus, effective 3′UTR-based NMD may rely not only on sensitivity to NMD but also insensitivity to other decay pathways. These observations, together with previous identification of RNA-binding proteins capable of preventing NMD of transcripts with long 3′UTRs [80, 81], suggest a complex set of determinants for decay of transcripts with long 3′UTRs and highlight the importance of considering competing decay pathways when drawing mechanistic conclusions from transcriptome-wide RNA abundance correlations.

Our data show that NR-seq and isoform-level EZbakR analyses can reveal distinct kinetic signatures of various RNA decay pathways and explain how different turnover processes interact to control basal RNA stability. Looking ahead, EZbakR analyses of NR-seq data can be used to dissect the kinetic mechanisms of many gene expression regulatory processes. For example, integration of EZbakR with RNA-binding protein (RBP) perturbation studies can identify whether specific RBPs modulate splicing decisions, transcript stability, or both. Applying isoform-level NR-seq to diverse cell types and disease models will help distinguish tissue-specific regulation from pathological disruptions in RNA processing, providing insights into disease mechanisms. These advances in understanding isoform-specific degradation kinetics also have practical applications, as they promise to improve our understanding of the nucleotide sequence determinants of RNA stability, knowledge that could guide the rational design of stable therapeutic mRNAs for gene therapy and RNA-based medicines.

## Conclusions

In this work, we expand the EZbakR-suite to enable isoform-level kinetic inference from short-read NR-seq data and introduce a strategy for refining and filtering transcript annotations, which is critical for accurately quantifying kinetic rate constants. Using the EZbakR-suite and acute NMD inhibition, we generate the first isoform-resolution kinetic map of human NMD. Our analyses uncover unexpected variability in the NMD efficiency of conserved poison exon-containing transcripts, the efficient targeting of non-PTC isoforms by NMD, and the impact of competing RNA decay pathways on both the regulatory and quality control functions of NMD. This study illustrates how isoform-level analyses with the EZbakR-suite can deepen our understanding of RNA kinetic regulation and provides a framework for investigating transcript-specific degradation kinetics, paving the way for future studies on how decay pathways shape the human transcriptome.

## Methods

### Small-molecule SMG1i synthesis

The small-molecule SMG1 kinase inhibitor (11j) was synthesized as previously described [26].

### SMG1i-treatment and TimeLapse chemistry

Human HEK-293 cells were obtained directly from ATCC and cultured at 37 °C under 5% carbon dioxide in a humidified chamber in Dulbecco’s modified Eagle medium supplemented with 10% v/v fetal bovine serum and 1% v/v penicillin–streptomycin. Cells tested negative for mycoplasma contamination and were not further authenticated in our laboratory. Cells were seeded at 0.3 × 10⁶ cells per well in eight wells across two 6-well plates the day before treatment. Cells were treated with either 1 µM SMG1i (four wells) or DMSO (four wells) for eight hours. Additionally, 100 µM s^4^U (Cayman Chemical 16373) was added to three wells from each condition for the last two hours. Media was removed, and total RNA was extracted using TRIzol Reagent. The TimeLapse workflow was then followed as previously described [14, 82].

### rRNA depletion for TimeLapse RNA-seq libraries

rRNA depletion of 1 µg total RNA was performed as previously described [83]. For library preparation, 100 ng of ribodepleted RNA was used as input for the NEBNext Ultra II Directional RNA Library Prep Kit (E7760). Libraries were prepared following the manufacturer's instructions, with an 8-min fragmentation time. Resulting sequencing libraries were approximately 450 bp in length. Libraries were sequenced by Admera Health, on the Illumina NovaSeq X + platform at a depth of 120 M reads per sample.

### PacBio full-length transcript sequencing (Iso-Seq)

PacBio Iso-Seq was performed by CD Genomics. A total of 2μg RNA was used as input for RNA sample preparation. First-strand cDNA synthesis, including reverse transcription and template switching, was carried out using the Clontech SMARTer™ PCR cDNA Synthesis Kit and the PacBio Iso-Seq Express Oligo Kit, followed by PCR amplification. SMRTbell libraries were generated using the SMRTbell Template Prep Kit. Sequencing was performed on single RNA samples from SMG1i and DMSO treatments, with a total read depth of 20 million reads. RNA sequencing data processing was carried out by first converting the BAM files into FASTQ format using the bam2fastq (Pacific Biosciences) tool for both DMSO and SMG1i samples. The resulting FASTQ files were aligned to the hg38 reference genome using minimap2 [84] with the splice-aware option, producing SAM files [*minimap2 -ax splice -ub hg38.fa ccs.fastq.gz* > *ccs.sam*]. The resulting SAM files were then converted to sorted BAM files using SAMtools [85] [*samtools view -bS ccs.sam | samtools sort -o ccs_sorted.bam*].

### AnnotationCleaner pipeline

AnnotationCleaner is a Snakemake pipeline that uses StringTie2 [40] in conjunction with custom scripting to build and refine custom annotations. It supports the use of short and long-read RNA-seq data. StringTie2 is used to assemble annotations for each sample (or pair of related short- and long-read samples), as well as to merge the individual assemblies. The merged assembly is then split into exonic and intronic bins, and featureCounts [86] is used to quantify the coverage of each of these bins (sizes of these bins can be adjusted; default used in this paper was for exonic bins to be 200 nt in length and intronic bins to be 600 nt in length). Exonic bins cover regions that are exonic in at least one annotated isoform, and intronic bins cover regions that are intronic in all annotated isoforms.

The intronic coverage of each gene is computed (in units of RPKM), and noisy estimates are regularized using genome-wide trends. More specifically, a regression model of exonic coverage vs. intronic coverage is fit, and the trendline is used as an informative prior to regularize gene-specific estimates. This specific strategy is identical to the replicate variability regularization strategy implemented in bakR [87] and EZbakR [18].

The coverage of each exonic bin is compared to the intronic coverage via a negative binomial null hypothesis test, and the resulting *p*-value is multiple-test adjusted using the method of Benjamini and Hochberg. Ends are trimmed by removing terminal exonic bins deemed poorly supported, and isoforms with unsupported exonic bins whose removal would introduce new splice junctions are left untrimmed and flagged in the final GTF file output by AnnotationCleaner (boolean column added called “problematic”).

### Isoform CDS and PTC annotations

The factR2 [88] package was used to characterize alternative splicing events, annotate CDSs, and predict isoforms containing PTCs. A factRObject was created from the transcript annotation file (SR-only.gtf or Mix.gtf), using the human genome as the reference. Annotated Ensembl CDSs were matched to transcripts in the custom annotation. The factR2-processed annotation was then passed to SQANTI3 (v5.2.2) [89] for further identification of novel CDSs using the GeneMarkS-T (GMST) [90] algorithm, using GENCODE v37 as the reference annotation and hg38 as the genome reference. To ensure accuracy, gffread [91] was used to correct any GTF anomalies, and ORFanage [92] was employed to integrate annotated ORFs from factR2 and SQANTI3. Finally, the refined annotation was reprocessed through factR2 to identify additional PTC-containing isoforms from all factR2 and SQANTI3 identified CDSs.

### Transcript isoform kinetic parameter estimation

We have implemented a novel linear mixing model in EZbakR, designed to estimate isoform-specific kinetic parameter estimates. It accepts as input transcript isoform abundance estimates and fraction new estimates for each transcript equivalence class (TEC). The former can come from any isoform quantification tool (RSEM is implemented in fastq2EZbakR and was used in all analyses presented here), and the latter is most easily obtained from EZbakR’s EstimateFractions() function, used in this work (default settings except pold_from_nolabel was set to TRUE, features was set to c(“XF”, “TEC”), and filter_condition was set to “|”). For each gene, the EstimateIsoformFractions() function fits the following linear mixing model using the method of maximum likelihood:

$$\theta_{\mathrm{TEC},i} \sim \mathrm{Beta}(\alpha, \beta)$$

$$\alpha = \overline{{\theta}_{\mathrm{TEC},i}} * \mathrm{nreads}_{i}$$

$$\beta = \mathrm{nreads}_i - \bar{\theta}_{\mathrm{TEC},i} * \mathrm{nreads}_i$$

$$\overline{\theta_{\mathrm{TEC},1}} = \sum_{j=1}^{N_i} p_j * \theta_{\mathrm{Isoprom},j}$$

$$p_j = \frac{\mathrm{TPM}_j}{\sum_{k=1}^{N_i} \mathrm{TPM}_k}$$

$${\theta}_{\mathrm{T}\mathrm{E}\mathrm{C},\mathrm{i}}= \text{ith TEC fraction new}$$

$$\overline{{\theta }_{\mathrm{T}\mathrm{E}\mathrm{C},\mathrm{i}}}= \text{fraction new of the ith TEC expected from isoform fraction new estimates}$$

$${\theta}_{\mathrm{I}\mathrm{s}\mathrm{o}\mathrm{f}\mathrm{o}\mathrm{r}\mathrm{m}, \mathrm{j}}= \text{estimated fraction new for jth isoform in the relevant TEC}$$

$${p}_{j}= \text{proportion of reads expected to be from jth isoform}$$

$${\mathrm{n}\mathrm{r}\mathrm{e}\mathrm{a}\mathrm{d}\mathrm{s}}_{i}= \text{number of reads from ith TEC}$$

$$\mathrm{T}\mathrm{P}\mathrm{M} = \text{estimated isoform abundance in transcripts per million}$$

Uncertainties in isoform fraction new estimates are obtained from the Hessian matrix. Isoform degradation and synthesis rate constants are then estimated from isoform fraction new values as follows:

$$k_{\mathrm{deg}} = - \frac{\ln(1 - \theta_{\mathrm{Isoform}})}{t_{\mathrm{label}}}$$

$$k_{\mathrm{syn}} = \mathrm{TPM} * k_{\mathrm{deg}}$$

We note that a limitation of this approach is that it requires assuming that RNA levels are at steady-state during the labeling [93]. This assumption can be violated as cells actively respond to SMG1i treatment. For example, increases in RNA synthesis during or just before labeling can inflate the labeled-to-unlabeled ratio and therefore mimic faster turnover. Notably, this artifact typically drives inferred turnover changes in the opposite direction of observed expression changes, making it less likely to explain many apparent stabilization events associated with RNA upregulation (Additional file 1: Fig. S2B).

All analyses required isoforms to have > 1 TPM in the DMSO condition to ensure robust kinetic parameter estimation. Below this threshold, variability in abundance and labeling measurements limited accurate decay rate inference. We note that this requirement may exclude highly efficient NMD substrates with very low steady-state abundance when NMD is active. All isoform-level analyses in Fig. 2 made use of the RefSeq annotation. Isoform analysis from Fig. 4 onward used the Mix annotation from AnnotationCleaner.

### Simulating NR-seq data

NR-seq data was simulated using NRsim, a Snakemake pipeline we developed (https://github.com/isaacvock/NRsim) [94]. NRsim combines polyester’s simulation of standard RNA-seq data with custom scripting to add T-to-C mutations to simulated sequencing reads in accordance with simulated kinetic parameter distributions and label times [95]. The input to NRsim is a real RNA-seq FASTQ file (or pair of FASTQ files), an annotation of transcript isoforms, and a genome FASTA file. The real data is used to estimate abundances of isoforms in the provided annotation using Salmon [96]. A filtered annotation is generated, removing isoforms with an estimated TPM < 1, and polyester is provided with this filtered annotation as well as the estimated isoform abundances. Kinetic parameters of synthesis and degradation are randomly drawn for each major transcript isoform (i.e., the most highly expressed isoform from each gene), with (by default) a 50% chance of differential isoform turnover explaining differences in alternative isoform abundance for the minor isoforms. Each read from a given isoform is then randomly assigned the status of “new” or “old” with probability equal to the simulated fraction new (calculated from the simulated degradation rate constant). Each T in a new read has a user-specified probability of being converted to a C, referred to as the “pnew”. The final FASTQ files are shuffled so as to not interfere with isoform abundance parameter estimation via tools like RSEM.

For the simulations in Fig. 1 and Additional file 1: Fig. S1, the RefSeq annotation and a SMG1i-treated replicate were provided to NRsim. For the simulations in Additional file 1: Fig. S4, RefSeq trimmed by AnnotationCleaner was provided instead, so that we could test analyzing the simulated data with the untrimmed and unfiltered RefSeq annotation.

### Reanalysis of UPF1-AID SLAM-seq and SMG7 knockout RNA-seq datasets

Reads from the E-MTAB-13836 [51, 97] were reprocessed as described above for the SMG1i dataset (Additional file 2: Table S3). Reads from the E-MTAB-9330 [66, 98] datasets were trimmed using fastp [99]. The trimmed reads were then aligned to the hg38 reference genome and Mix transcript annotation using default STAR [100] parameters. Isoform quantification was performed using RSEM [20], and differential expression analysis was conducted using limma/voom via the Degust server [101, 102] (Additional file 2: Table S7). To assess the relationship between *SMG7* knockout effects and changes observed in our SMG1i dataset, we performed separate linear regression analyses for PTC-containing and non-PTC transcript groups. Transcripts with missing values were excluded prior to analysis. Within each group, we fit a linear model regressing SMG1i L2FC(k_deg_) on SMG7KO L2FC(RNA) values and extracted the correlation coefficient (R) and its associated *p*-value.

### Isoform Decay Range (IDR) analysis

The Isoform Decay Range (IDR) was determined using the output of EZbakR’s AverageAndRegularize() function. For each multi-isoform gene, IDR was calculated as the difference in the replicate average log(k_deg_) between the least and most stable isoforms under a given condition (DMSO or SMG1i) (Additional file 2: Table S4). Statistical significance was assessed using a two-tailed z-test, comparing the ratio of the range to the total uncertainty in the range against a standard normal distribution. To compare IDRs between DMSO- and SMG1i-treated cells, the IDR difference served as the test statistic numerator, while the denominator represented the total uncertainty of this difference. *p*-values were adjusted for multiple testing using the Benjamini–Hochberg method.

### Conserved poison exon analysis

Potential alternative splicing events were extracted from the Mix annotation GTF using the SUPPA2 generateEvents function [103]. Junctions evaluated by EZbakR were assigned to SUPPA splicing events and integrated with factR2 PTC predictions [88]. Coordinates corresponding to inclusion and exclusion junctions of conserved poison exons were obtained from [57] and converted to hg38 coordinates using the UCSC liftover tool [104]. Conserved poison exons selected for further analysis were those that had exact matches to at least one inclusion and one exclusion coordinate of SUPPA2-identified splicing events and were predicted to contain PTCs by factR2.

### PARE-seq reanalysis

Reads from the GSE61398 [63, 105] dataset were trimmed using fastp [99] [-L -b 20]. The trimmed reads were then aligned to the hg38 reference genome using STAR [100] [–sjdbOverhang 19]. PARE peaks were called and read coverage was quantified using the CAGEfightR [106] package, with a coverage cutoff of 1 TPM per cluster and a cluster merge distance of 20 nucleotides. The Degust [101] server was used to TMM normalize cluster coverage. Differential coverage was evaluated using RUV-seq [107], applying the RUVr option (k = 1). The highest coverage positions within each cluster were mapped to the Mix transcript annotation coordinates using the GenomicFeatures [108] and RCAS [109] packages. Clusters mapping to positions between −10 and + 50 relative to TCs and significantly depleted in SMG6 or UPF1 knockdown conditions (L2FC < −1 and FDR < 0.05) were selected for further visual inspection.

### *XRN1* and *SMG6* knockdowns and Akron5-seq library preparation

Human HEK-293T cells were obtained directly from ATCC and cultured at 37 °C under 5% carbon dioxide in a humidified chamber in Dulbecco’s modified Eagle medium supplemented with 10% v/v fetal bovine serum and 1% v/v penicillin–streptomycin. Cells tested negative for mycoplasma contamination and were not further authenticated in our laboratory. For depletion of XRN1 and SMG6, HEK-293T cells were seeded at 3 × 105 cells per well and reverse transfected with 40 nM siRNA targeting *XRN1* (Forward: 5′ AGAUGAACUUACCGUAGAA 3′; Reverse: 5′ UUCUACGGUAAGUUCAUCU 3′) or *SMG6* (Forward: 5′ GCUGCAGGUUACUUACAAG 3′; Reverse: 5′ CUUGUAAGUAACCUGCAGC 3′) [110], or a negative control (Negative Control #2, Thermo Scientific) using Lipofectamine RNAiMAX per manufacturer instructions. After 48h, cells were replated at 5 × 105 cells per well in a 6-well plate. The next day, total RNA was extracted using TRIzol (Invitrogen) with overnight isopropanol precipitation at −80 °C. RNA was then DNase-treated (dsDNase, Thermo Scientific) and purified with 0.6 × SPRIselect beads (Beckman Coulter).

Three biological replicates were processed per condition. A total of 150 µg RNA per sample was used to generate Akron libraries, as described previously [62], with modifications. Total RNA underwent two rounds of poly(A) selection using the NEB Magnetic mRNA Isolation Kit and 100 µL oligo(dT) beads. RNA was denatured for 90 s at 80 °C, incubated with beads for 15 min at room temperature, and sequentially washed with 1 × Bind/Wash Buffer (5 mM Tris–HCl pH 7.5, 0.5 mM EDTA, 1 M NaCl), followed by Wash Buffer B (10 mM Tris–HCl pH 7.5, 1 mM EDTA, 150 mM LiCl) and nuclease-free water.

Poly(A)-enriched mRNA was ligated to a 0.2 µM 5′ biotin-tagged RNA adaptor using T4 RNA Ligase (Thermo Scientific) at 16 °C, 800 rpm, overnight. The following day, after three washes with Bind/Wash buffer, mRNA was fragmented using 10 × Fragmentation Reagent (Thermo Scientific) for 7 min at 70 °C. Fragmented mRNA was purified using Dynabeads M-280 streptavidin beads (Invitrogen) in a 1:1 ratio for 30 min at room temperature. After washing, end repair was performed for 1 h at 37 °C using T4 PNK (NEB). The mRNA was then ligated to a 0.1 µM 3′ adaptor at 16 °C, 800 rpm, overnight. The 3′ adaptor contained a 5ʹ phosphate group to enhance ligation efficiency, a 3ʹ-dideoxycytidine to prevent unwanted ligation, and a 10-nucleotide unique molecular identifier (UMI) for PCR deduplication.

cDNA synthesis was performed using SuperScript III Reverse Transcriptase (Invitrogen) and an RT primer (sequence listed in table below), followed by two rounds of PCR amplification using NEBNext Ultra II Q5 Master Mix. The first PCR used DP5 and RT primers (16 cycles), while the second PCR used the 5′ FP universal primer and NEB index primers (8 cycles) under the following conditions: 98 °C for 30 s, followed by cycles of 98 °C for 10 s and 65 °C for 75 s, and a final extension at 65 °C for 5 min. PCR products were ethanol-precipitated overnight at −80 °C with 2.5 × ethanol, 0.1 × 3 M sodium acetate pH 5.5, and GlycoBlue. Size selection was performed using Pippin Prep (1.5% agarose, marker K, Sage Science) to isolate fragments between 500–800 bp. Final libraries were quantified using the Quant-IT PicoGreen dsDNA Kit (Promega), analyzed on an Agilent Tapestation 2200 with DS1000 screentape, and sequenced on an Illumina NovaSeq X plus at a depth of > 30 million reads per library.

**Table:** 

Name	Sequence
5′ Biotin RNA Adaptor	/5Biosg/rGrUrUrCrArGrArGrUrUrCrUrArCrArGrUrCrCrGrArCrGrArUrC
3′ 5Phos and 3ddc adaptor	/5Phos/NNNNNNNNNNAGATCGGAAGAGCACACGTCT/3ddC/
RT primer	AGACGTGTGCTCTTCCGATCT
DP5	GTTCAGAGTTCTACAGTCCGACGATC
5′ FP universal primer	AATGATACGGCGACCACCGAGATCTACACGTTCAGAGTTCTACAGTCCGA

### Akron5-seq data processing and analysis

UMIs were extracted from sequencing reads using UMI-tools [111]. Reads were subsequently quality-trimmed using fastp [99] with automatic adapter detection and poly-G tail removal. Trimmed reads were mapped to Repbase [112] using STAR [100] (v2.7.6a) with up to 30 allowed multimapping locations. Unmapped reads were subsequently aligned to the hg38 reference genome using STAR with parameters optimized for accurate splice junction detection and paired-end overlap resolution. Uniquely mapping reads were extracted using samtools [85] (q = 255) and deduplicated based on unique molecular identifiers (UMIs) with UMI-tools. Only properly paired, non-chimeric alignments were retained. To quantify putative SMG6 cleavage sites, 5′ end read alignments were extracted from deduplicated BAM files using samtools and converted into BigWig tracks with CAGEfightR [106]. Cleavage clusters were identified using CAGEfightR with a threshold of 1 TPM and merging distances of 20 nucleotides. Cleavage site counts were assigned to transcript features using the Mix annotation and exported. Differential cluster abundance analysis was performed using Degust [101]. Cleavage clusters with > 10 counts in at least three samples were normalized with TMM, and batch effects were corrected using RUVr [107] (k = 2). Differentially abundant clusters were identified with edgeR maximum likelihood estimation. Clusters mapping to positions between −10 and + 50 relative to non-PTC isoform TCs, significantly depleted in *SMG6* knockdown conditions (L2FC < −1 and FDR < 0.05), and significantly stabilized by SMG1i (L2FC(k_deg_) < −0.25, padj < 0.05) were selected for further visual inspection. See Additional file 2: Table S5 for analysis output.

### LASSO regression and classification models

The glmnet R package was used to train LASSO regression models with our data [113, 114]. We trained two types of models in this work: a degradation rate constant regression model and a SMG1i sensitivity classification model. In both cases, feature engineering was performed to make LASSO regression coefficients comparable across features. Numeric features were standardized (subtracted the mean and divided by the standard deviation), with numeric features ranging many orders of base-10 magnitude being log_10_ transformed prior to standardization. Engineering categorical features is more difficult. We settled on a strategy that involved ordering categorical features based on their average outcome variable value (e.g., lowest to highest degradation rate constant). For categorical features with more than 10 groups, we pooled groups with similar average outcome variables. This is to avoid artificially strong correlation between the ranks of a categorical feature and the outcome due to super-fine stratification of the data. We then z-scored the ranks assigned to each group and used that as the engineered feature. While this approach still risks artificially inflating the importance assigned to categorical features, we note that categorical features were never identified as the strongest predictors in any of our models. We also only used feature importances as inspiration for downstream analyses to more carefully probe the impact that these features may be having.

For the degradation rate constant regression model, we only considered transcript isoforms with high confidence estimates. Thus, we filtered out low coverage isoforms, isoforms with high degradation rate constant estimate uncertainty, and isoforms flagged by either of our filtering criteria (Fig. 3A). We then trained the model with 100 different bootstrapped samples of the engineered features and DMSO-treated degradation rate constant estimates, to assess stability of estimated feature importances. An optimal lambda value was determined through 10-fold cross validation on each bootstrapped dataset.

For the SMG1i sensitivity classification model, we only considered transcript isoforms which we could confidently assign the status of SMG1i sensitive or not. Thus, we filtered out low coverage isoforms and isoforms with high degradation rate constant estimate uncertainty. High confidence SMG1i-sensitive isoforms were the remaining isoforms that had a log_2_-fold change in their degradation rate constant of less than −0.7, and with a degradation rate constant after SMG1i inhibition less than 65% of its value prior to SMG1i inhibition (suggesting at least around 35% of its degradation is attributable to NMD). High confidence SMG1i insensitive isoforms were those with a log_2_-fold change in their degradation rate constant of around 0 (or that had estimated increases in their rate constant). For the classification model, we did not filter out unsupported isoforms or those from high JCC genes, as we were using a reduced set of isoforms to begin with and suspected that comparative analyses of degradation rate constant estimates were more robust to annotation discrepancies than single-condition estimates. To deal with the large class imbalance, we downsampled the insensitive isoform set to the size of the sensitive set. Feature importances were estimated for 100 different downsamplings to assess stability of these estimates. An optimal lambda value was determined through 10-fold cross validation on each downsampled dataset. The model was evaluated on held-out test data from all bootstrapped samples. Receiver operating characteristic and precision recall curves, as well as the areas under these curves, were obtained with the pROC [115] and yardstick [116] libraries in R, respectively.

Features used in these analyses are provided in Additional file 2: Table S8. Briefly, sequence features were calculated from the Mix annotation file using custom R and Python scripts. Additional data, such as RF1 and RF2 peptide release rates [117] and codon optimality [118], were either directly merged or processed before integration, as detailed below.

### CLIP-UPF1 affinity purification-seq reanalysis

Reads from the GSE134059 [69, 119] dataset were trimmed using fastp [99] with parameters [-L -b 20]. The trimmed reads were then aligned to the hg38 reference genome and Mix transcript annotation using default STAR [100] parameters. Isoform quantification was performed using RSEM [20], and differential expression analysis of CLIP-UPF1 vs GFP AP was conducted using limma/voom via the Degust server [101, 102].

### TC-containing exon PhastCons scoring

To identify exons containing stop codons, a GTF file was processed using the R package GenomicRanges [108]. Exon entries were filtered, and stop codon positions were determined from the Mix transcript annotation. Exons overlapping stop codons were extracted and converted to BED format. PhastCons conservation scores were then obtained from the UCSC hg38 100-way alignment using the phastCons100way.UCSC.hg38 (Bioconductor) package. The BED file was imported as a GenomicRanges object, and PhastCons scores were computed for the selected exons.

### Isoform Kozak sequence scoring

A custom python script was used to assess the strength of Kozak sequences in the Mix transcript annotation FASTA file. A position-specific scoring matrix [120, 121] was defined based on nucleotide positions within the 10-base Kozak sequence. For each sequence in the FASTA file, a Kozak score was computed by summing the matrix values for the corresponding nucleotides at each position.

## Supplementary Information

Additional file 1: Supplementary figures. Figs. S1–S8 provide supplementary analyses supporting Figs. 1, 2, 3, 4, 5, 6 and 7, including simulation benchmarks, annotation-quality analyses, NMD-regulation analyses, Akron5-seq/PARE-seq validation, and LASSO model evaluation. Fig. S1. Simulated isoform-level NR-seq analyses. Fig. S2. Evaluation of isoform-level NR-seq analyses. Fig. S3. Examples of spurious annotations resolved by custom annotation assembly and curation. Fig. S4. Evaluation of annotations and validation of k_deg_ estimates. Fig. S5. Comparison of differential transcript stability upon SMG1i treatment and UPF1 depletion. Fig. S6. Features associated with intrinsic transcript instability affecting NMD efficiency. Fig. S7. Identification of EJC-independent substrates. Fig. S8. Performance and results of machine learning analyses revealing key features of PTC and non-PTC isoform-specific NMD kinetics.

Additional file 2: Supplementary tables. Tables S1–S8 provide alignment statistics, EZbakR outputs, UPF1-AID and *SMG7*-knockout reanalyses, isoform decay range estimates, Akron5-seq outputs, junction-level analyses, and LASSO model features/metrics. Table S1. STAR alignment statistics and EZbakR labeling information. Table S2. EZbakR isoform-level outputs from Ensembl, RefSeq, SRonly, and Mix annotations. Table S3. EZbakR isoform-level reanalysis of the UPF1-AID dataset using the Mix annotation. Table S4. Isoform decay range estimates. Table S5. Analysis of Akron5-seq data. Table S6. EZbakR junction-level descriptions and outputs. Table S7. Isoform-level reanalysis of SMG7-KO datasets using the Mix annotation. Table S8. Features included in the LASSO regression models, model metrics for all isoforms, and model metrics for PTC isoforms.

## Data Availability

All datasets are included in the main References list and cited in the text. Short-read NR-seq FASTQ and processed bedgraph files and long-read BAM files are publicly available in GEO under accession GSE289489 [122], and short-read Akron5-seq FASTQ and processed BigWig files are publicly available in GEO under accession GSE291379 [123]. All original code is publicly available on GitHub [124]. Previously published datasets used in this study are available under accession numbers GSE207924 [21], GSE143662 [22], GSE61398 [105], GSE134059 [119], E-MTAB-13836 [97], and E-MTAB-9330 [98], and are cited in the main text and Methods and included in the references list.
