# Supplementary material for: Uncovering the isoform-resolution kinetic landscape of nonsense-mediated mRNA decay with EZbakR
Source: Genome Biol. 2026 Jul 15;27:313. doi: 10.1186/s13059-026-04204-w (PMC13651330; doi:10.1186/s13059-026-04204-w)
Supplement: Supplementary file 1 [file 13059_2026_4204_MOESM1_ESM.pdf]

Additional File 1: Supplementary figures

Fig. S1

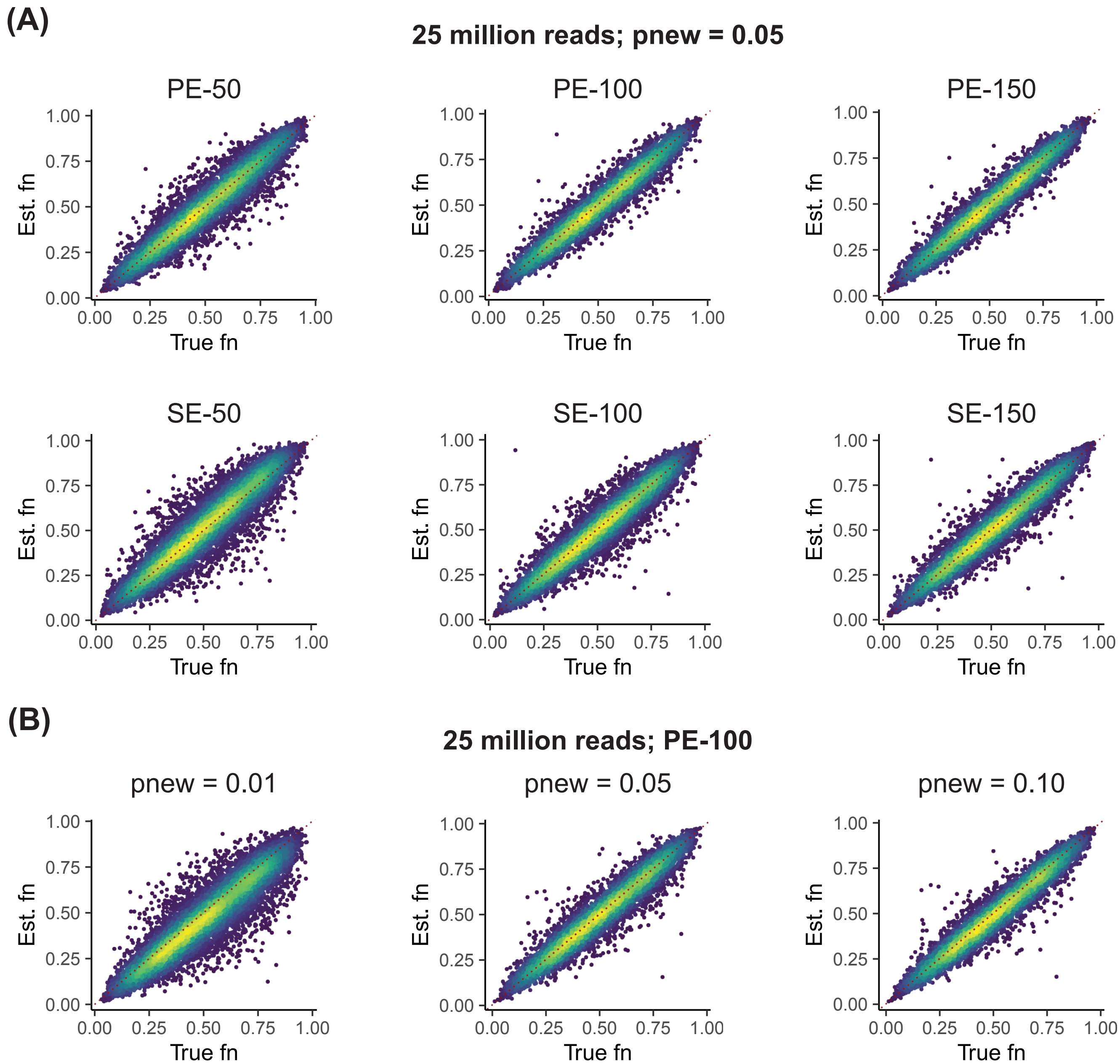

Fig. S1. Simulated isoform-level NR-seq analyses, associated with Fig. 1
(A) Further simulated data assessment of isoform fraction new estimate accuracy across a range of read lengths. PE denotes paired-end data and SE denotes single-end data. Points are colored by density, and the red dotted line represents perfect estimation (y = x). **(B)** Further simulated data assessment of isoform fraction new estimate accuracy across a range of incorporation rates (pnew; defined as the rate of T-to-C conversion in reads from new RNA). Points are colored by density, and the red dotted line represents perfect estimation (y = x).

Fig. S2

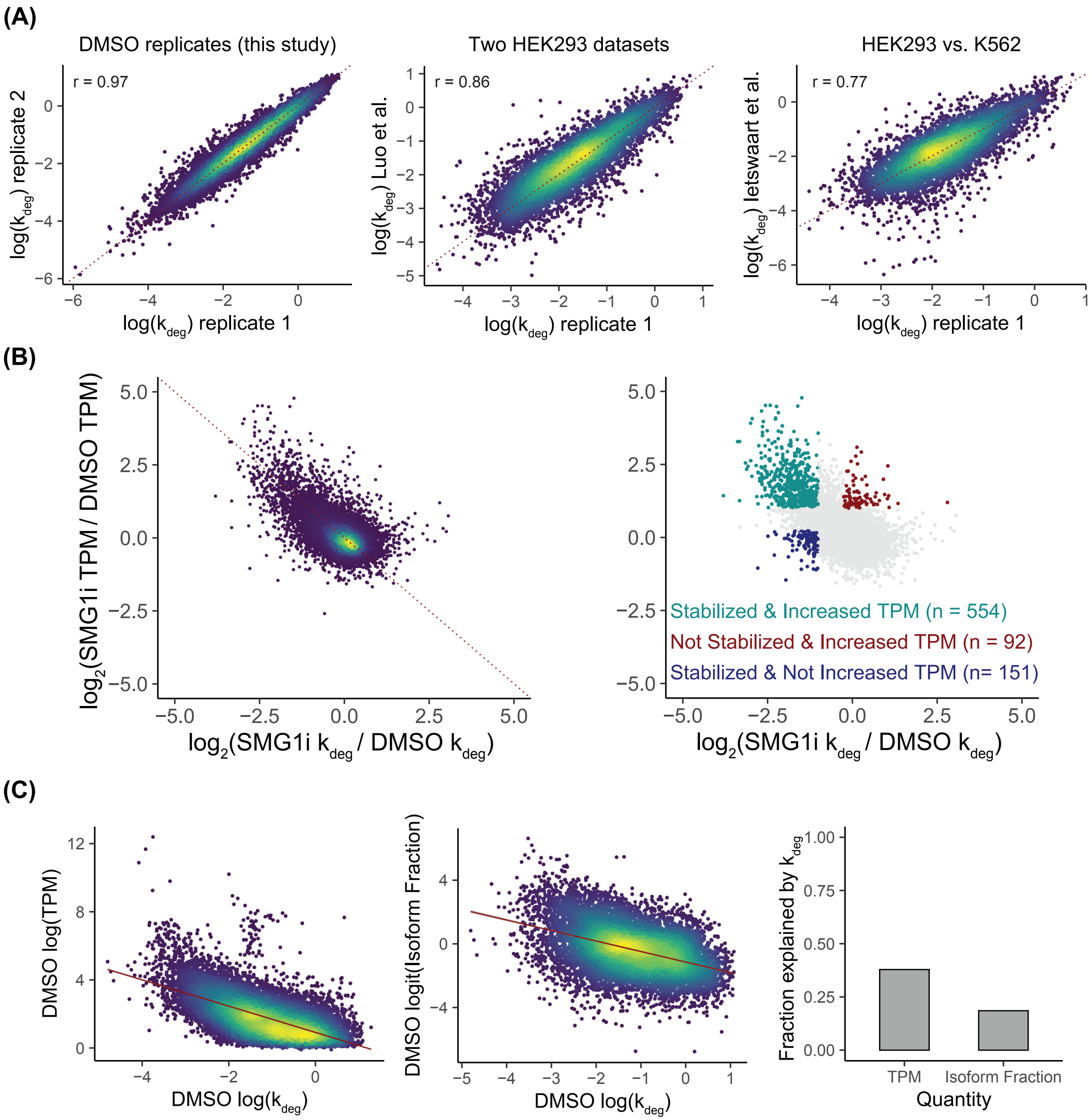
(A)
DMSO replicates (this study)
Two HEK293 datasets
HEK293 vs. K562
r = 0.97
r = 0.86
r = 0.77
log(k_deg) replicate 2
log(k_deg) Luo et al.
log(k_deg) letswaart et al.
log(k_deg) replicate 1
(B)
log2(SMG1i TPM / DMSO TPM)
log2(SMG1i k_deg / DMSO k_deg)
Stabilized & Increased TPM (n = 554)
Not Stabilized & Increased TPM (n = 92)
Stabilized & Not Increased TPM (n= 151)
(C)
DMSO log(TPM)
DMSO log(k_deg)
DMSO logit(Isoform Fraction)
Fraction explained by k_deg
TPM
Isoform Fraction
Quantity

Fig. S2. Evaluation of isoform-level NR-seq analyses, associated with Fig. 2
(A) Consistency of EZbakR isoform-level degradation rate constant estimates. Left: comparison of two replicates of DMSO-treated HEK-293 cell degradation rate constant estimates from this study. Middle: comparison of one replicate of DMSO-treated HEK-293 cell degradation rate constant estimates from this study to those from one replicate of WT HEK-293 cell from a previously published dataset [22,24]. Right: comparison of one replicate of DMSO-treated HEK-293 cell degradation rate constant estimates from this study to those from one replicate of WT K562 cells from a previously published dataset [21,23]. **(B)** Extent to which differential stability explains differential expression upon SMG1 inhibition. Both plots compare the changes in degradation rate constant upon SMG1 inhibition (x-axis) to the change in estimated isoform abundance upon SMG1 inhibition (y-axis). Left: points colored by density. Right: highlights three sets of isoforms: 1) those that are both strongly stabilized (L2FC(k_{deg}) < -1) and strongly upregulated (L2FC(TPM) > 1); 2) those that are not strongly stabilized (L2FC(k_{deg}) > -0.25) but are strongly upregulated; and 3) those that are strongly stabilized but not strongly upregulated (L2FC(TPM) < 0.25). **(C)** Extent to which isoform stability explains isoform abundance. **Left**: comparison of log-scale transcript isoform degradation rate constant in DMSO-treated cells (x-axis) to its abundance (y-axis). The red line represents the linear best fit. **Middle**: comparison of the transcript isoform degradation rate constant (x-axis) to its isoform fraction (the fraction of transcripts from a gene contributed by a specific isoform) on a logodds (logit) scale (y-axis). The red line is the linear best fit. **Right**: coefficient of determination for the linear regressions shown in the Left and Middle plots. The y-axis is thus interpretable as the fraction of isoform-to-isoform variance in either of the two y-axis quantities in those plots that is explained by the transcript isoform degradation rate constant.

Fig. S3

(A)

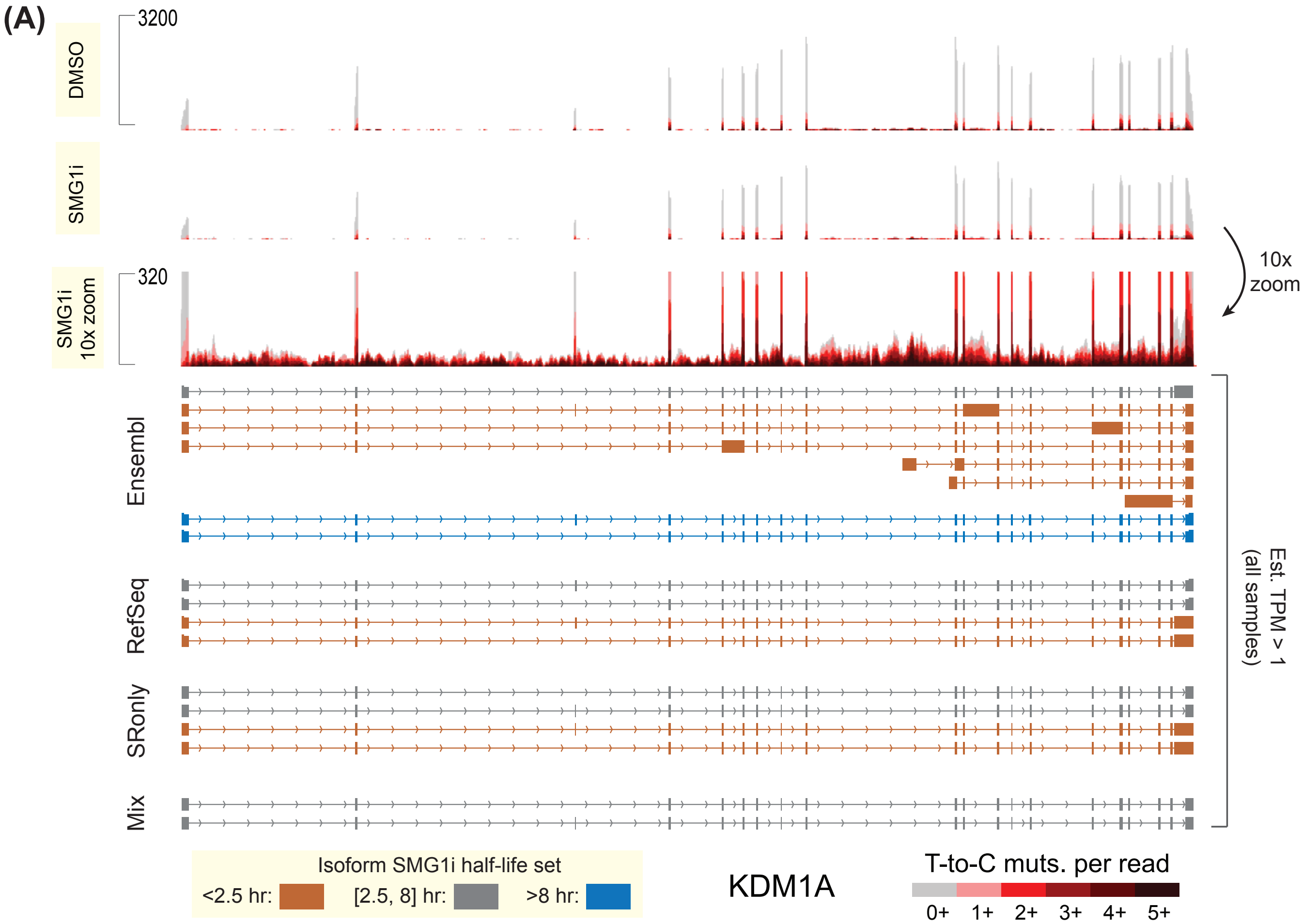

3200
DMSO
SMG1i
320
SMG1i 10x zoom
10x zoom
Ensembl
RefSeq
SRonly
Mix
Est. TPM > 1 (all samples)
Isoform SMG1i half-life set
<2.5 hr:
[2.5, 8] hr:
>8 hr:
KDM1A
T-to-C muts. per read
0+
1+
2+
3+
4+
5+

(B)

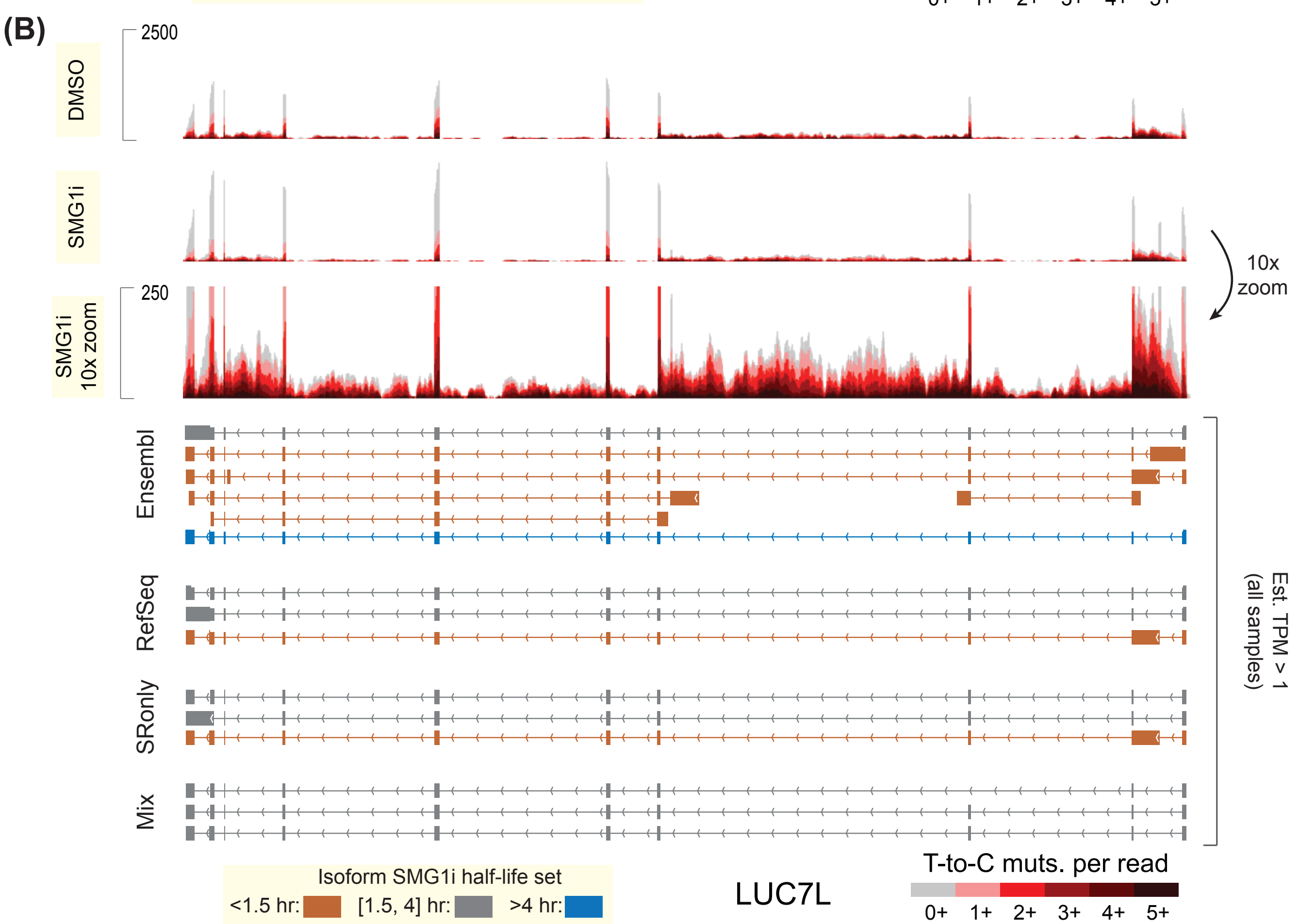

2500
DMSO
SMG1i
250
SMG1i 10x zoom
10x zoom
Ensembl
RefSeq
SRonly
Mix
Est. TPM > 1 (all samples)
Isoform SMG1i half-life set
<1.5 hr:
[1.5, 4] hr:
>4 hr:
LUC7L
T-to-C muts. per read
0+
1+
2+
3+
4+
5+

Fig. S3. Examples of spurious annotations resolved by custom annotation assembly and curation, associated with Fig. 3

(A) TimeLapse-seq sequencing tracks for *KDM1A* and the 4 annotations discussed in this work. Mix indicates the annotation built using both short-read and long-read RNA-seq data, and SRonly indicates the annotation built using only short-read data. **Top track**: A representative replicate of DMSO-treated data. **Middle track**: A representative replicate of SMG1i-treated data. **Bottom track**: Middle track with an approximately 10x zoomed vertical scale to show intronic coverage and mutational content. Annotations: Isoform maps below tracks show isoforms with an RSEM estimated abundance of > 1 TPM in all samples. Isoforms are colored by their half-life, with isoforms binned into unstable (< 2.5 hour), moderately stable (between 2.5 and 8 hour), and highly stable (> 8 hour) transcripts. Note both the preponderance of highly unstable transcripts (orange isoform models) in the Ensembl annotation as well as the extent to which the presence of these isoforms inflates some estimates of stable isoform turnover (blue isoform models). Spurious annotations tend to include intronic regions with a high fraction of labeled reads, leading to frequent errors in decay rate constant estimation when using Ensembl annotations (see also Fig. 3D and Fig. S4B). **(B)** Same as (A) but for *LUC7L*. Transcript half-life set bounds are adjusted to better reflect the range of turnover kinetics at this locus.

Fig. S4

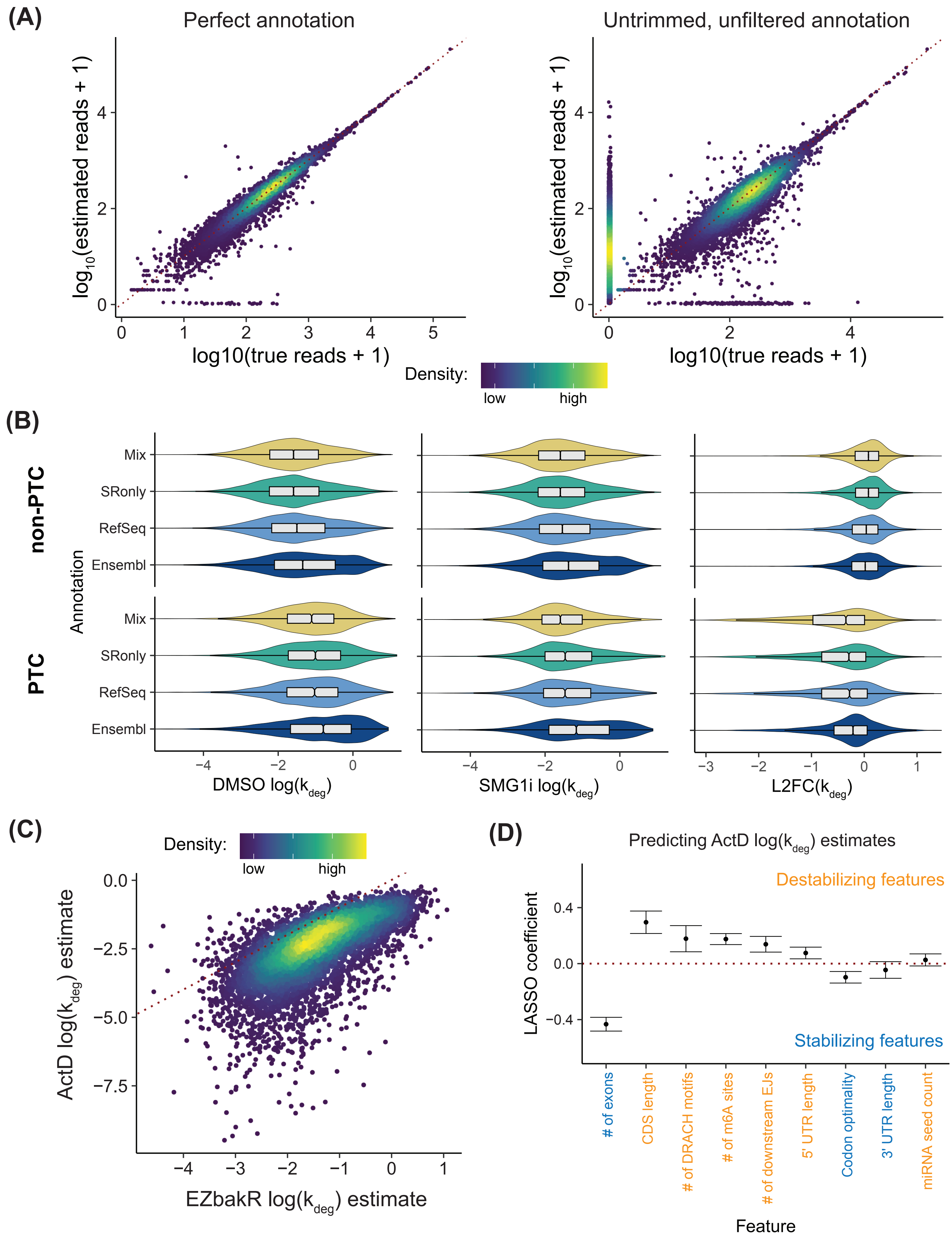

(A)
Perfect annotation
Untrimmed, unfiltered annotation
log10(estimated reads + 1)
log10(true reads + 1)
Density:
low
high
(B)
non-PTC
PTC
Annotation
Mix
SRonly
RefSeq
Ensembl
DMSO log(kdeg)
SMG1i log(kdeg)
L2FC(kdeg)
(C)
Density:
low
high
ActD log(kdeg) estimate
EZbakR log(kdeg) estimate
(D)
Predicting ActD log(kdeg) estimates
Destabilizing features
Stabilizing features
LASSO coefficient
# of exons
CDS length
# of DRACH motifs
# of m6A sites
# of downstream EJs
5' UTR length
Codon optimality
3' UTR length
miRNA seed count
Feature

Fig. S4. Evaluation of annotations and validation of k_{deg} estimates, associated with Fig. 3. **(A)** Assessment of transcript isoform quantification accuracy with simulated data and the use of different annotations. **Left**: Simulated data analyzed using the exact annotation from which the simulated data was generated. Same as what is used in Fig. 1 and S1. **Right**: Same simulated data but analyzed with an annotation including an amount of UTR overextension and number of unexpressed isoforms reflective of that seen in real data. Points are colored by density. Note the large number of reads spuriously assigned (points at x = 0) in simulations using the untrimmed, unfiltered annotation. **(B)** Comparison of turnover kinetic trends seen in the 4 annotations assessed. **Left**: log-scale degradation rate constants in DMSO-treated cells (x-axis) for all isoforms with estimated TPM > 1 in all samples. **Middle**: Same as Left but for SMG1i-treated cells. **Right**: Changes in degradation rate constants upon SMG1i treatment for all isoforms with estimated TPM > 1 in all samples. Note qualitatively different distributions of kinetic parameters when using Ensembl annotations. **(C)** Comparison of NR-seq/EZbakR derived isoform degradation rate constant estimates to those obtained from transcript isoform quantification of a previously published transcription inhibition RNA-seq time course, also in HEK-293 cells [50]. Points are colored by density. **(D)** LASSO regression coefficients for the same analysis shown in Fig. 3F, but with log-scale degradation rate constants estimated from the transcription inhibition RNA-seq time course rather than our TimeLapse-seq data.

Fig. S5

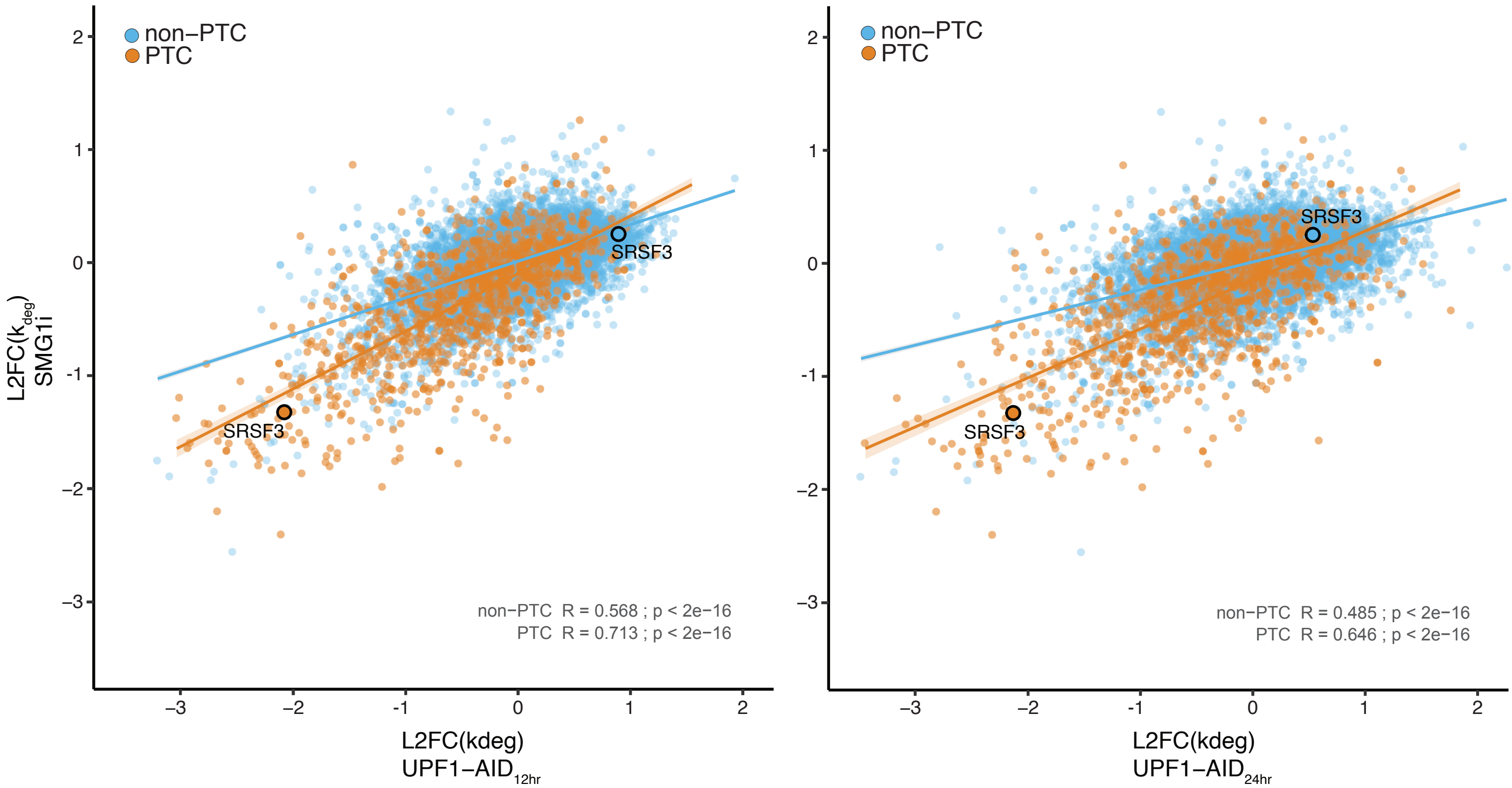
non−PTC
PTC
L2FC(k_deg)
SMG1i
SRSF3
SRSF3
non−PTC R = 0.568 ; p < 2e−16
PTC R = 0.713 ; p < 2e−16
L2FC(kdeg)
UPF1−AID12hr
non−PTC
PTC
SRSF3
SRSF3
non−PTC R = 0.485 ; p < 2e−16
PTC R = 0.646 ; p < 2e−16
L2FC(kdeg)
UPF1−AID24hr

Fig. S5. Comparison of differential transcript stability upon SMG1i treatment and UPF1 depletion, associated with Fig. 4.

Scatter plots comparing SMG1i L2FC(k_{deg}) with UPF1-AID L2FC(k_{deg}) [51] after 12 hour (**left**) and 24 hour (**right**) depletions, shown separately for non-PTC (blue) and PTC-containing (orange) isoforms. Linear regression lines with standard error shading are displayed for each group, along with the corresponding Pearson correlation coefficients (R) and p-values. *SRSF3* isoforms are discussed in the text.

Fig. S6

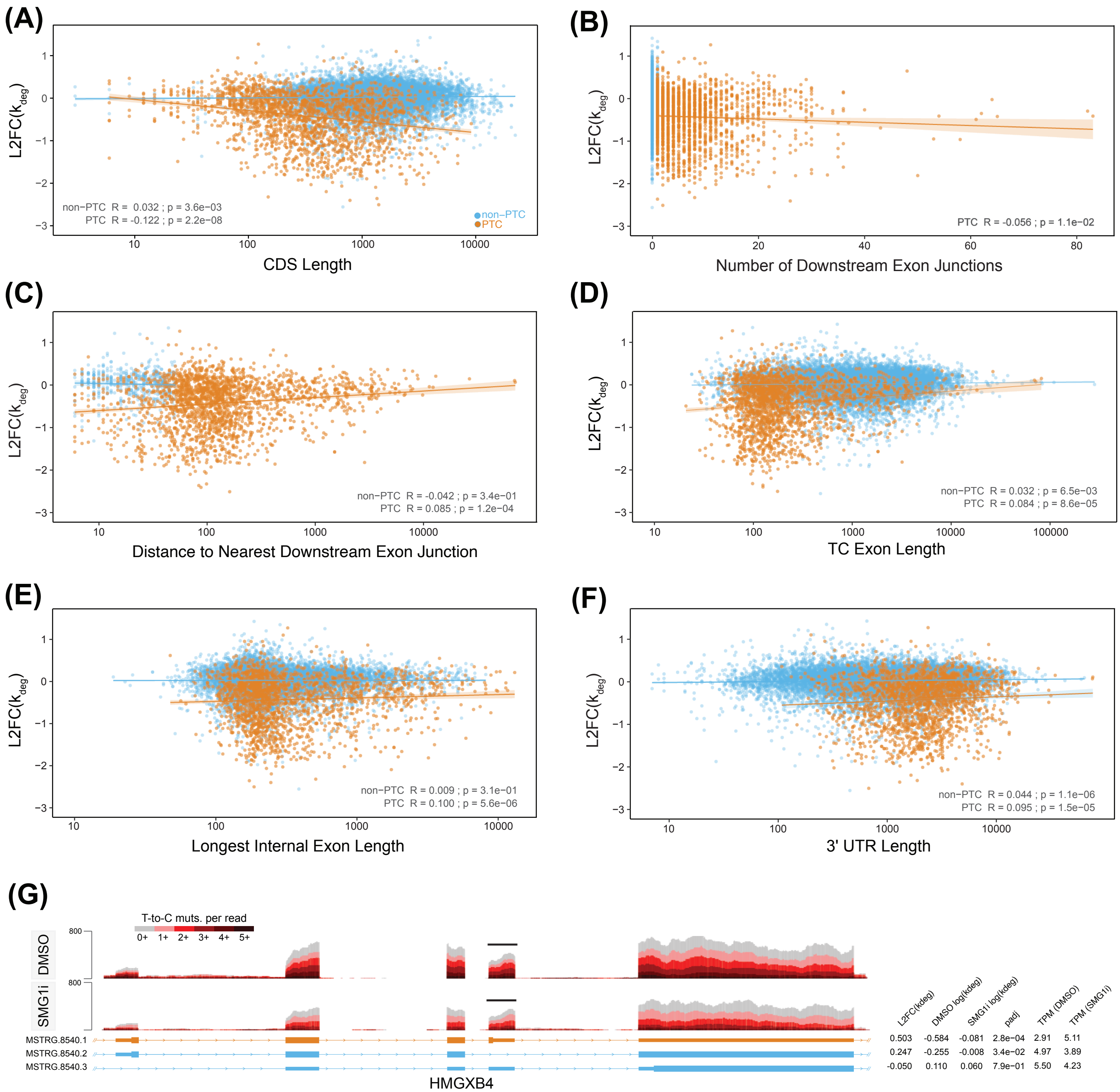
(A)
L2FC(kdeg)
non−PTC R = 0.032 ; p = 3.6e−03
PTC R = -0.122 ; p = 2.2e−08
non−PTC
PTC
CDS Length
(B)
PTC R = -0.056 ; p = 1.1e−02
Number of Downstream Exon Junctions
(C)
non−PTC R = -0.042 ; p = 3.4e−01
PTC R = 0.085 ; p = 1.2e−04
Distance to Nearest Downstream Exon Junction
(D)
non−PTC R = 0.032 ; p = 6.5e−03
PTC R = 0.084 ; p = 8.6e−05
TC Exon Length
(E)
non−PTC R = 0.009 ; p = 3.1e−01
PTC R = 0.100 ; p = 5.6e−06
Longest Internal Exon Length
(F)
non−PTC R = 0.044 ; p = 1.1e−06
PTC R = 0.095 ; p = 1.5e−05
3' UTR Length
(G)
T-to-C muts. per read
0+ 1+ 2+ 3+ 4+ 5+
DMSO
SMG1i
L2FC(kdeg) DMSO log(kdeg) SMG1i log(kdeg) padj TPM (DMSO) TPM (SMG1i)
MSTRG.8540.1 0.503 -0.584 -0.081 2.8e-04 2.91 5.11
MSTRG.8540.2 0.247 -0.255 -0.008 3.4e-02 4.97 3.89
MSTRG.8540.3 -0.050 0.110 0.060 7.9e-01 5.50 4.23
HMGXB4

Fig. S6. Features associated with intrinsic transcript instability affecting NMD efficiency, associated with Fig. 5.

(A-F) Scatter plots showing the log2-fold change in the isoform degradation rate constant (L2FC(k_{deg})) between SMG1i and DMSO treatments as a function of **(A)** CDS length, **(B)** number of downstream exon junctions, **(C)** distance to nearest downstream exon junction, **(D)** TC exon length, **(E)** longest internal exon length, and **(F)** 3′UTR length for non-PTC (blue) and PTC-containing (orange) isoforms. Linear regression fit lines with standard error shading are shown for PTC data, along with corresponding correlation coefficients (R) and significance values (p-value) for each regression. **(G)** TimeLapse-seq tracks for the HMGXB4 gene under DMSO (upper tracks) and SMG1i (lower tracks) treatment conditions. The degree of s4U labeling is represented by increasing darkness of red, corresponding to the number of T-to-C mutations per read, with unlabeled reads shown in gray. Three *HMGXB4* isoforms are schematized at the bottom, with the ORFs represented by thicker lines and the black bar indicating the position of the poison exon. Blue isoforms are non-PTC isoforms, the orange isoform is PTC-containing. Decay kinetics and RNA metrics for these isoforms are provided on the right.

Fig. S7

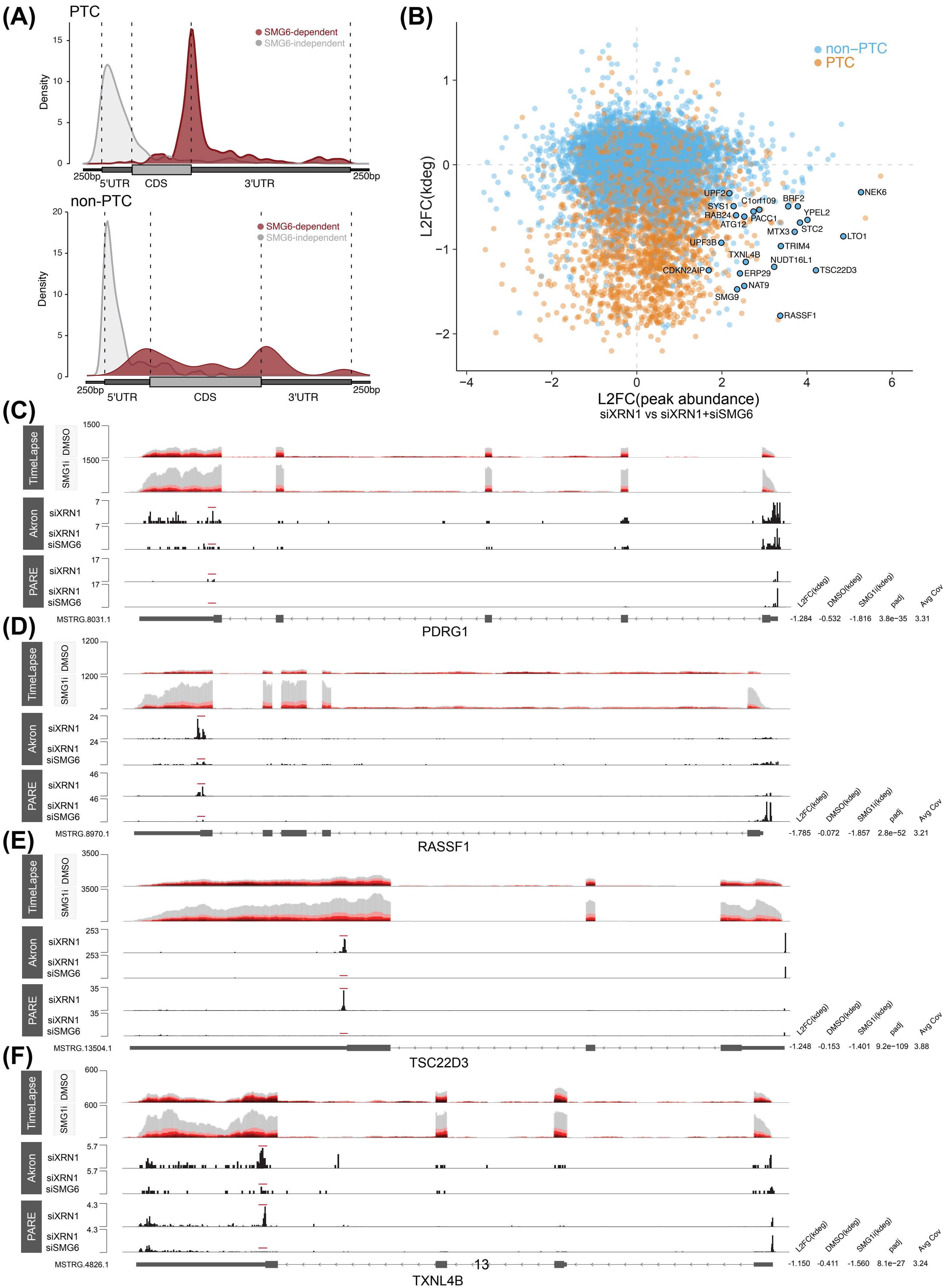
(A)
PTC
SMG6-dependent
SMG6-independent
Density
250bp
5'UTR
CDS
3'UTR
250bp
non-PTC
(B)
non–PTC
PTC
L2FC(kdeg)
L2FC(peak abundance)
siXRN1 vs siXRN1+siSMG6
NEK6
LTO1
TSC22D3
RASSF1
(C)
TimeLapse
DMSO
SMG1i
Akron
PARE
siXRN1
siXRN1 siSMG6
MSTRG.8031.1
PDRG1
L2FC(kdeg)
DMSO(kdeg)
SMG1i(kdeg)
padj
Avg Cov
-1.284 -0.532 -1.816 3.8e-35 3.31
(D)
MSTRG.8970.1
RASSF1
-1.785 -0.072 -1.857 2.8e-52 3.21
(E)
MSTRG.13504.1
TSC22D3
-1.248 -0.153 -1.401 9.2e-109 3.88
(F)
MSTRG.4826.1
TXNL4B
-1.150 -0.411 -1.560 8.1e-27 3.24

Fig. S7

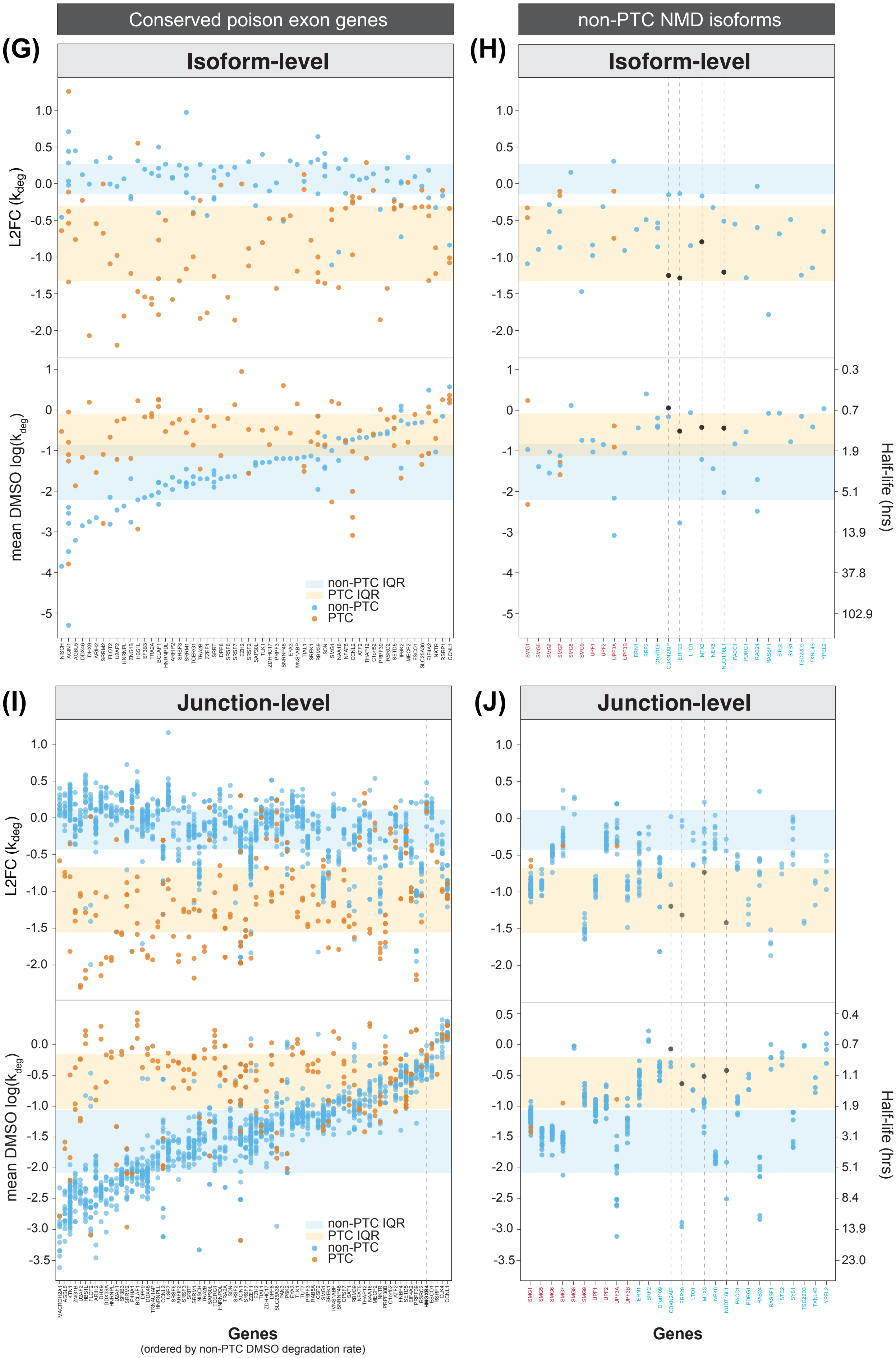

Conserved poison exon genes
non-PTC NMD isoforms
(G)
Isoform-level
(H)
Isoform-level
L2FC (k_deg)
mean DMSO log(k_deg)
Half-life (hrs)
non-PTC IQR
PTC IQR
non-PTC
PTC
(I)
Junction-level
(J)
Junction-level
Genes
(ordered by non-PTC DMSO degradation rate)
Genes

Fig. S7

(K)

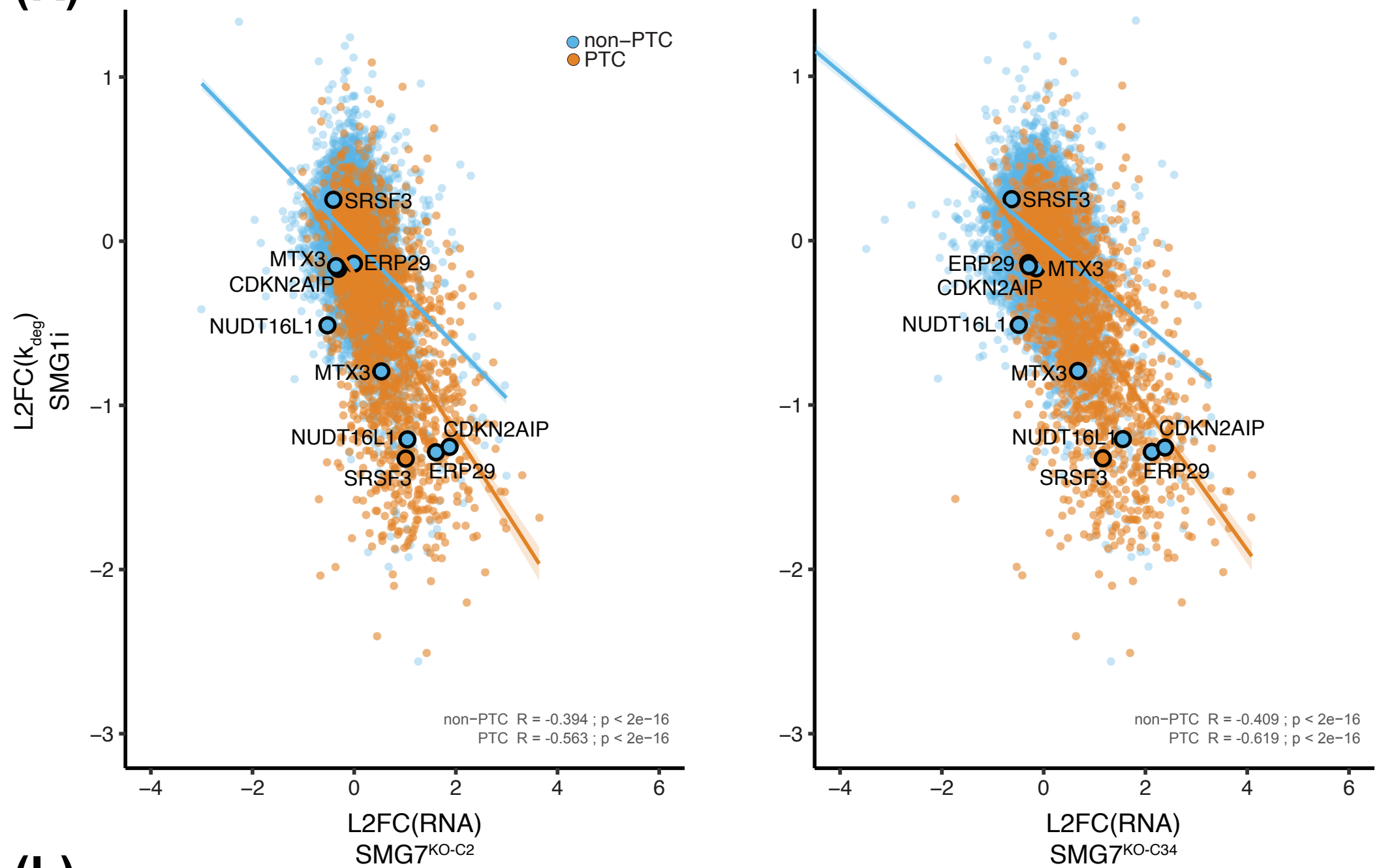

non−PTC
PTC
SRSF3
MTX3
ERP29
CDKN2AIP
NUDT16L1
MTX3
NUDT16L1
CDKN2AIP
SRSF3
ERP29
non−PTC R = -0.394 ; p < 2e−16
PTC R = -0.563 ; p < 2e−16
L2FC(k_deg)
SMG1i
L2FC(RNA)
SMG7KO-C2
SRSF3
ERP29
MTX3
CDKN2AIP
NUDT16L1
MTX3
NUDT16L1
CDKN2AIP
SRSF3
ERP29
non−PTC R = -0.409 ; p < 2e−16
PTC R = -0.619 ; p < 2e−16
L2FC(RNA)
SMG7KO-C34

(L)

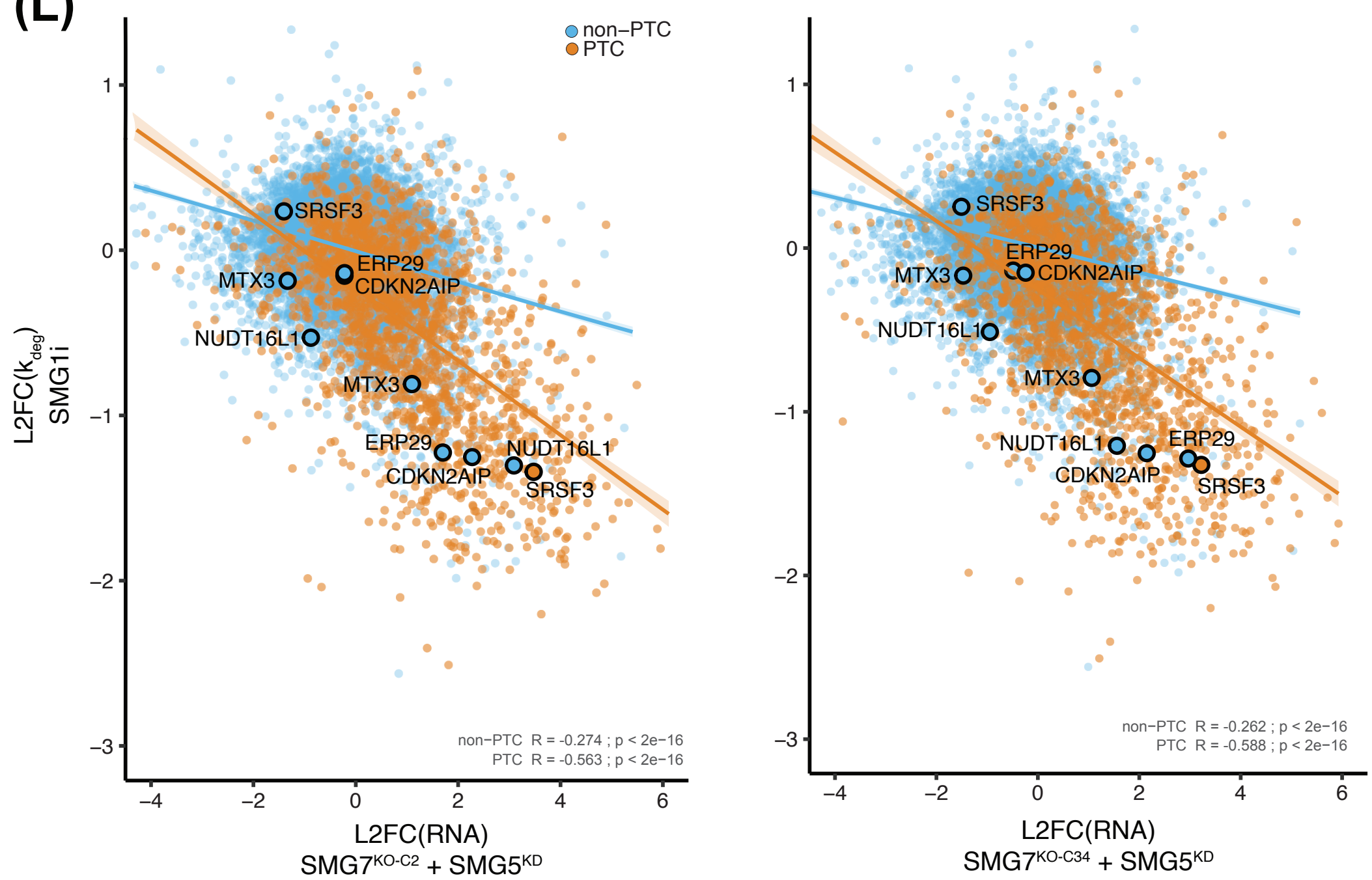

non−PTC
PTC
SRSF3
ERP29
MTX3
CDKN2AIP
NUDT16L1
MTX3
ERP29
NUDT16L1
CDKN2AIP
SRSF3
non−PTC R = -0.274 ; p < 2e−16
PTC R = -0.563 ; p < 2e−16
L2FC(k_deg)
SMG1i
L2FC(RNA)
SMG7KO-C2 + SMG5KD
SRSF3
ERP29
MTX3
CDKN2AIP
NUDT16L1
MTX3
NUDT16L1
ERP29
CDKN2AIP
SRSF3
non−PTC R = -0.262 ; p < 2e−16
PTC R = -0.588 ; p < 2e−16
L2FC(RNA)
SMG7KO-C34 + SMG5KD

Figure S7

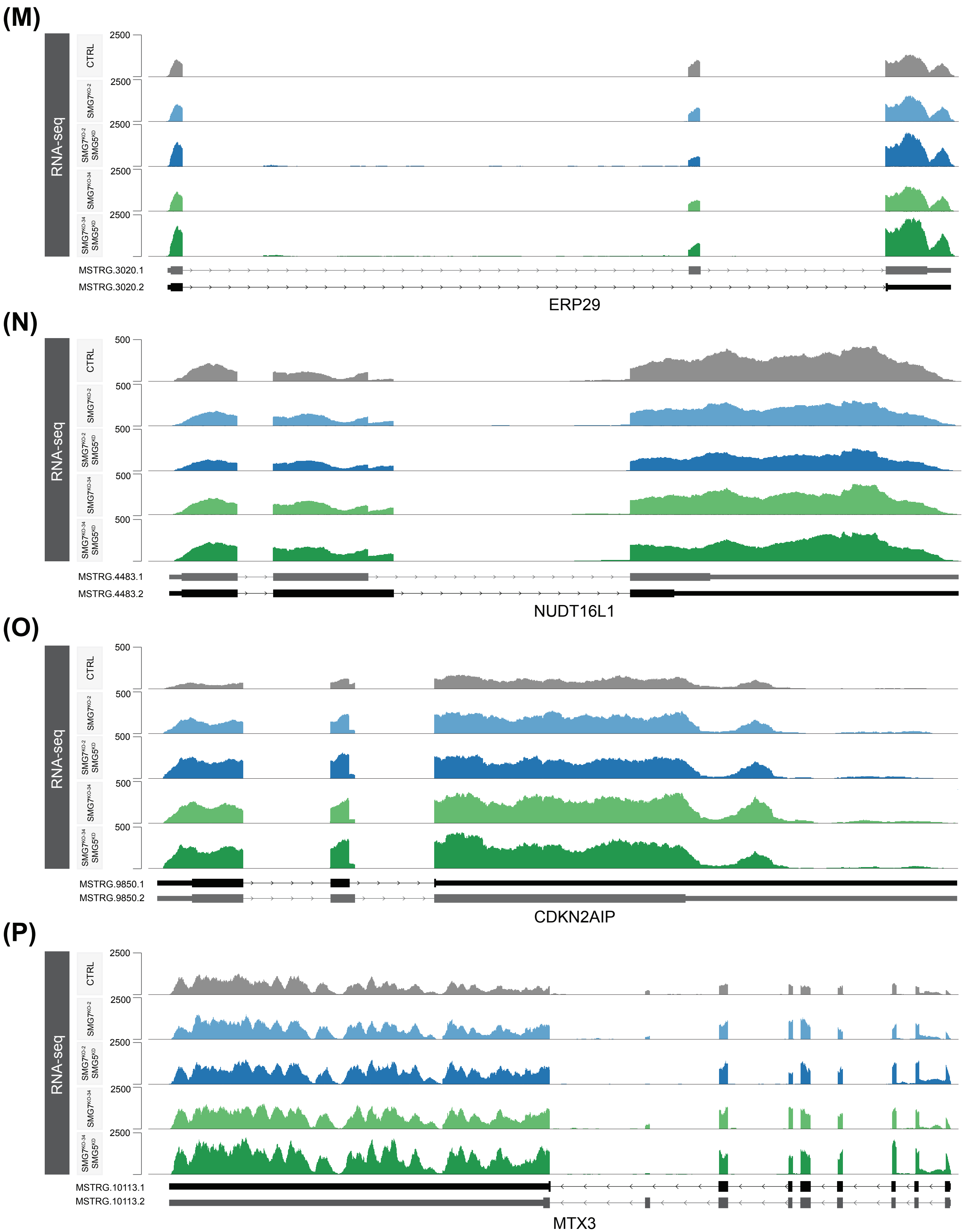
(M)
RNA-seq
CTRL
SMG7KO-2
SMG7KO-2 SMG5KD
SMG7KO-34
SMG7KO-34 SMG5KD
2500
MSTRG.3020.1
MSTRG.3020.2
ERP29
(N)
500
MSTRG.4483.1
MSTRG.4483.2
NUDT16L1
(O)
500
MSTRG.9850.1
MSTRG.9850.2
CDKN2AIP
(P)
2500
MSTRG.10113.1
MSTRG.10113.2
MTX3

Fig. S7. Identification of EJC-independent substrates, associated with Fig. 6.

(A) Meta-gene analysis showing the distribution of peaks with significantly increasing (red) or decreasing (gray) L2FC(peak abundance) in siXRN1 vs. siXRN1+siSMG6 conditions. The top panel represents PTC-containing isoforms, while the bottom panel represents non-PTC isoforms. Below each distribution is a schematic of a meta-transcript. Gray peaks primarily correspond to increased decapping resulting from *SMG6* knockdown, while red peaks indicate free 5′ phosphates resulting from SMG6-dependent cleavage events. **(B)** Scatter plot comparing isoform-level L2FC(peak abundance) between siXRN1 and siXRN1+siSMG6 conditions (x-axis) to L2FC(k_{deg}) between SMG1i and DMSO treatments (y-axis) for non-PTC (blue) and PTC-containing (orange) isoforms. Transcripts in the bottom right quadrant are highly stabilized by SMG1i treatment and show strong evidence of TC-proximal SMG6 cleavage. Genes highlighted in the figure are discussed further in the text and throughout Fig. S7. **(C-F) Top**: TimeLapse-seq tracks for *PDRG1* **(C)**, *RASSF1* **(D)**, *TSC22D3* **(E)** and *TXNL4B* **(F)** under DMSO (upper tracks) and SMG1i (lower tracks) treatment conditions. The degree of s4U labeling is indicated by increasing darkness of red, corresponding to the number of T-to-C mutations per read, with unlabeled reads shown in gray. **Bottom**: Akron5-seq (this study) and PARE-seq [63] tracks from *XRN1* and *XRN1+SMG6* depletion experiments, highlighting SMG6-dependent cleavage sites marked by red lines above the sequence tracks. The isoform from each gene is schematized below, with the ORFs represented by thicker lines. Corresponding decay rate measurements for these isoforms are displayed to the right. **(G)** Distribution of isoform-level stability measurements for conserved poison exon-containing genes. **Top**: Dot plots displaying the distribution of isoform-level L2FC(k_{deg}) changes between SMG1i and DMSO treatments for a set of conserved poison exon-containing genes. **Bottom**: Dot plots showing the distribution of isoform-level DMSO log(k_{deg}) decay rate measurements for the same set of genes. Genes are ordered by the DMSO degradation rate of their non-PTC isoforms, from lowest to highest. Blue dots represent non-PTC isoforms, and orange dots represent PTC-containing isoforms. The orange shaded region highlights the interquartile range (IQR) of L2FC(k_{deg}) changes in SMG1i vs. DMSO (top) or the mean DMSO k_{deg} values (bottom) for PTC-containing isoforms. Similarly, the blue shaded region indicates the IQR of L2FC(k_{deg}) changes in SMG1i vs. DMSO for non-PTC isoforms. **(H)** As in (G), but for NMD factor genes and others with NMD-sensitive non-PTC isoforms. The shaded regions highlight the IQRs of the L2FC(k_{deg}) in SMG1i vs. DMSO (top) or the mean DMSO k_{deg} values (bottom) for PTC-containing isoforms (orange) and non-PTC isoforms (blue) of poison exon-containing genes (same ranges as in G). Genes are color-coded, with NMD factors in red and high-confidence non-PTC isoforms in blue. **(I-J)** Junction-based stability measurements for validated PTC **(I)** and non-PTC **(J)** NMD-targeted genes. Top: Dot plots showing the distribution of junction-level L2FC(k_{deg}) in degradation rate measurements between SMG1i and DMSO treatments. Bottom: Dot plots showing the distribution of junction-level DMSO log(k_{deg}) degradation rate measurements for the same set of genes, ordered and colored as in (G) and (H). Blue dots represent non-PTC junctions, while orange dots represent junctions associated with PTC-exons. The orange shaded region highlights the IQR of L2FC(k_{deg}) changes in SMG1i vs. DMSO (top) or the mean DMSO log(k_{deg}) values (bottom) for PTC-containing isoforms of poison exon-containing genes. Similarly, the blue shaded region indicates the IQR of L2FC(k_{deg}) changes in SMG1i vs. DMSO for non-PTC isoforms of poison exon-containing genes. **(K)** Scatter plots comparing SMG1i L2FC(k_{deg}) with *SMG7* knockout L2FC(RNA) [66] for clone 2 (**left**) and clone 34 (**right**), shown separately for non-PTC (blue) and PTC-containing (orange) isoforms. Linear regression lines with standard error shading are displayed for each group, along with the corresponding Pearson correlation coefficients (R) and p-values. Genes highlighted in the figure are discussed further in the text. **(L)** Scatter plots comparing SMG1i L2FC(k_{deg}) with *SMG7* knockout L2FC(RNA) clone 2 (**left**) or clone 34 (**right**) in combination with *SMG5* knockdown, as described in (K). Highlighted genes are discussed in the text. **(M–P)** RNA-seq tracks for PTC-free AS-NMD genes *ERP29* (**M**), *NUDT16L1* (**N**), *CDKN2AIP* (**O**), and *MTX3* (**P**) under control conditions (upper track)

and *SMG7* knockout ± *SMG5* knockdown (lower tracks). The relevant isoform for each gene is schematized below, with open reading frames indicated by thicker lines. SMG1i-sensitive isoforms are shown in black, and insensitive isoforms in gray.

Fig. S8

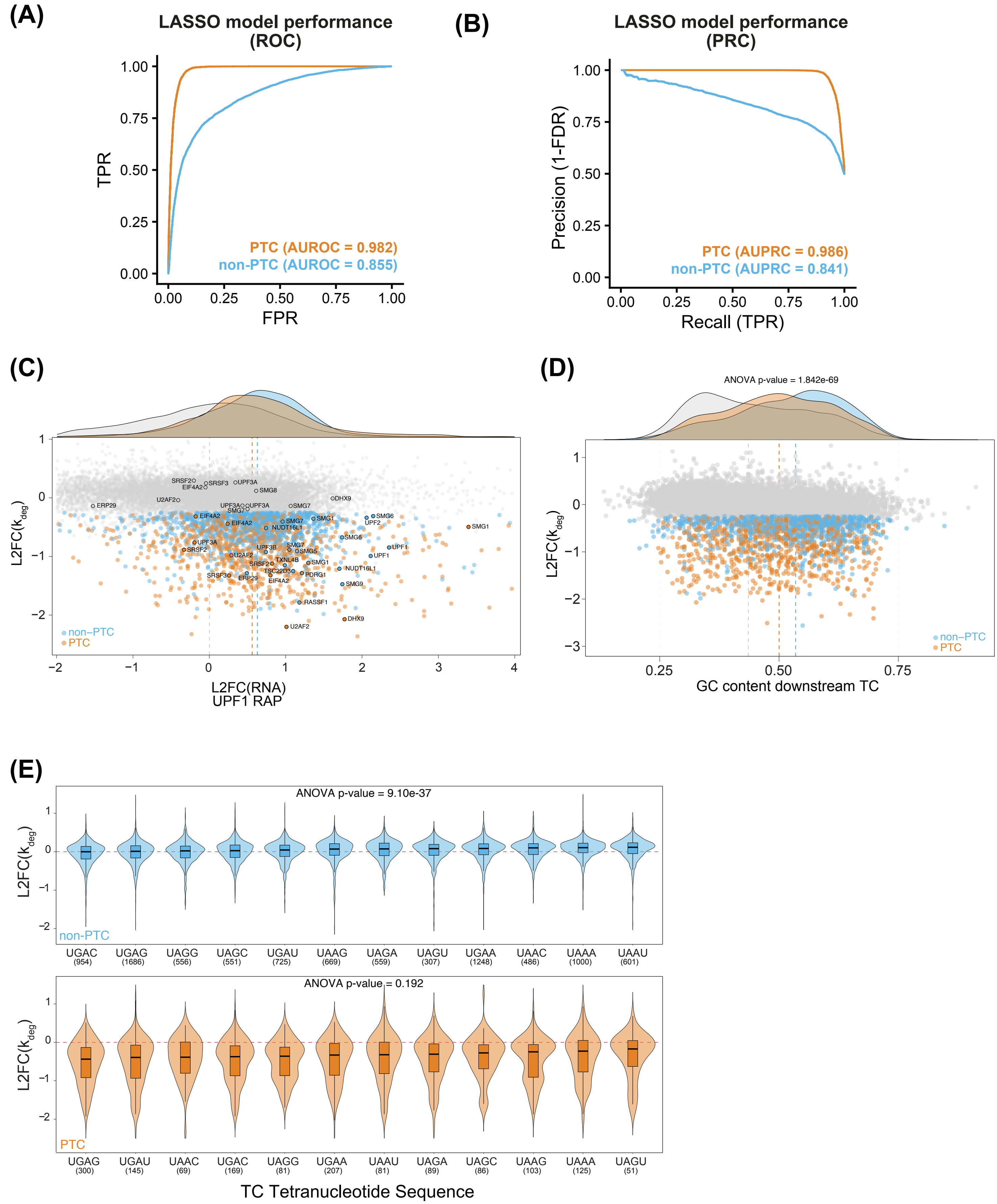

(A)
LASSO model performance (ROC)
TPR
FPR
PTC (AUROC = 0.982)
non-PTC (AUROC = 0.855)
(B)
LASSO model performance (PRC)
Precision (1-FDR)
Recall (TPR)
PTC (AUPRC = 0.986)
non-PTC (AUPRC = 0.841)
(C)
L2FC(k_deg)
L2FC(RNA) UPF1 RAP
non−PTC
PTC
(D)
ANOVA p-value = 1.842e-69
L2FC(k_deg)
GC content downstream TC
non−PTC
PTC
(E)
ANOVA p-value = 9.10e-37
L2FC(k_deg)
non-PTC
UGAC (954) UGAG (1686) UAGG (556) UAGC (551) UGAU (725) UAAG (669) UAGA (559) UAGU (307) UGAA (1248) UAAC (486) UAAA (1000) UAAU (601)
ANOVA p-value = 0.192
PTC
UGAG (300) UGAU (145) UAAC (69) UGAC (169) UAGG (81) UGAA (207) UAAU (81) UAGA (89) UAGC (86) UAAG (103) UAAA (125) UAGU (51)
TC Tetranucleotide Sequence

Fig. S8

(F)

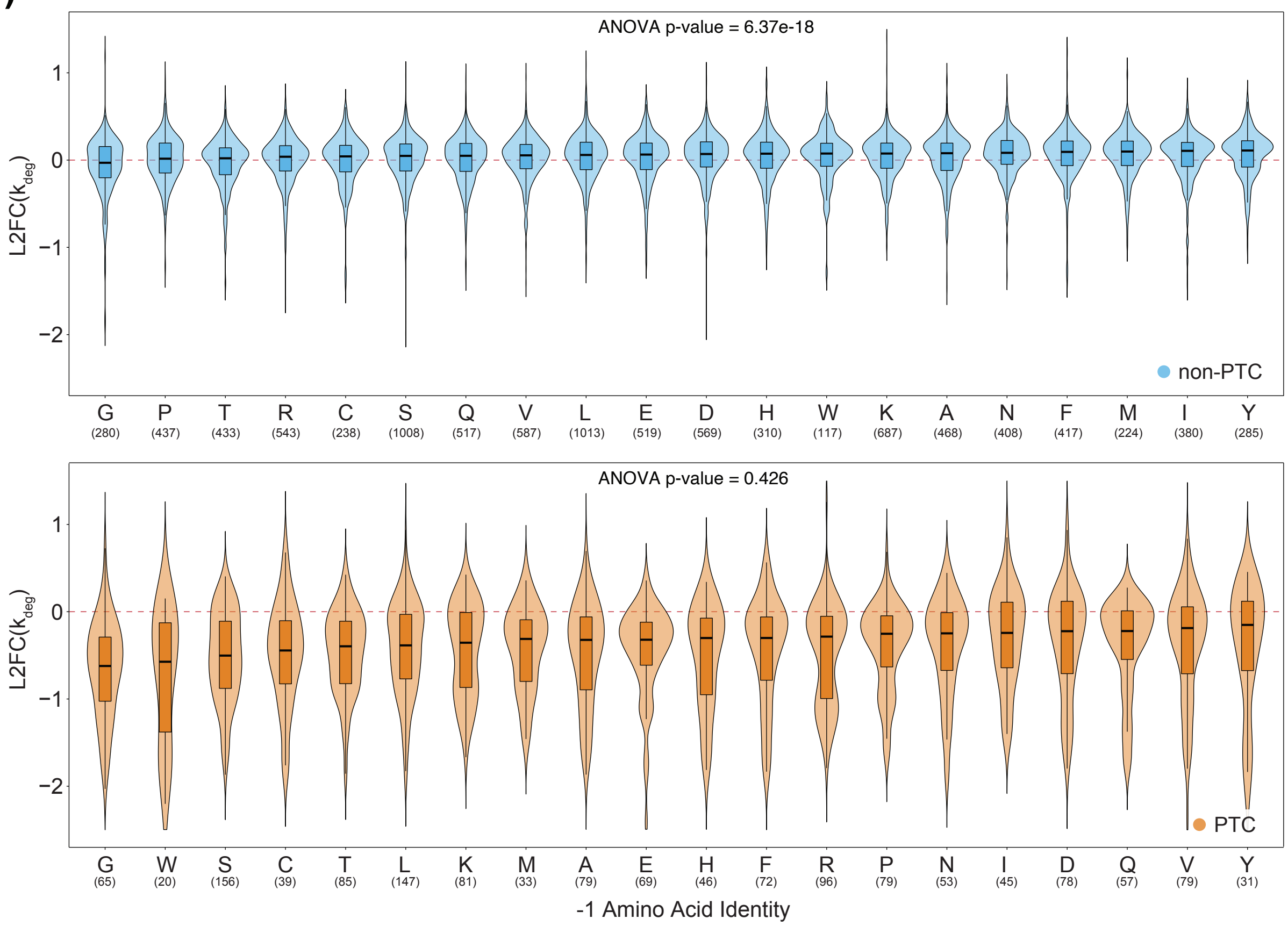
ANOVA p-value = 6.37e-18
L2FC(k_deg)
non-PTC
G (280) P (437) T (433) R (543) C (238) S (1008) Q (517) V (587) L (1013) E (519) D (569) H (310) W (117) K (687) A (468) N (408) F (417) M (224) I (380) Y (285)
ANOVA p-value = 0.426
PTC
G (65) W (20) S (156) C (39) T (85) L (147) K (81) M (33) A (79) E (69) H (46) F (72) R (96) P (79) N (53) I (45) D (78) Q (57) V (79) Y (31)
-1 Amino Acid Identity

(G)

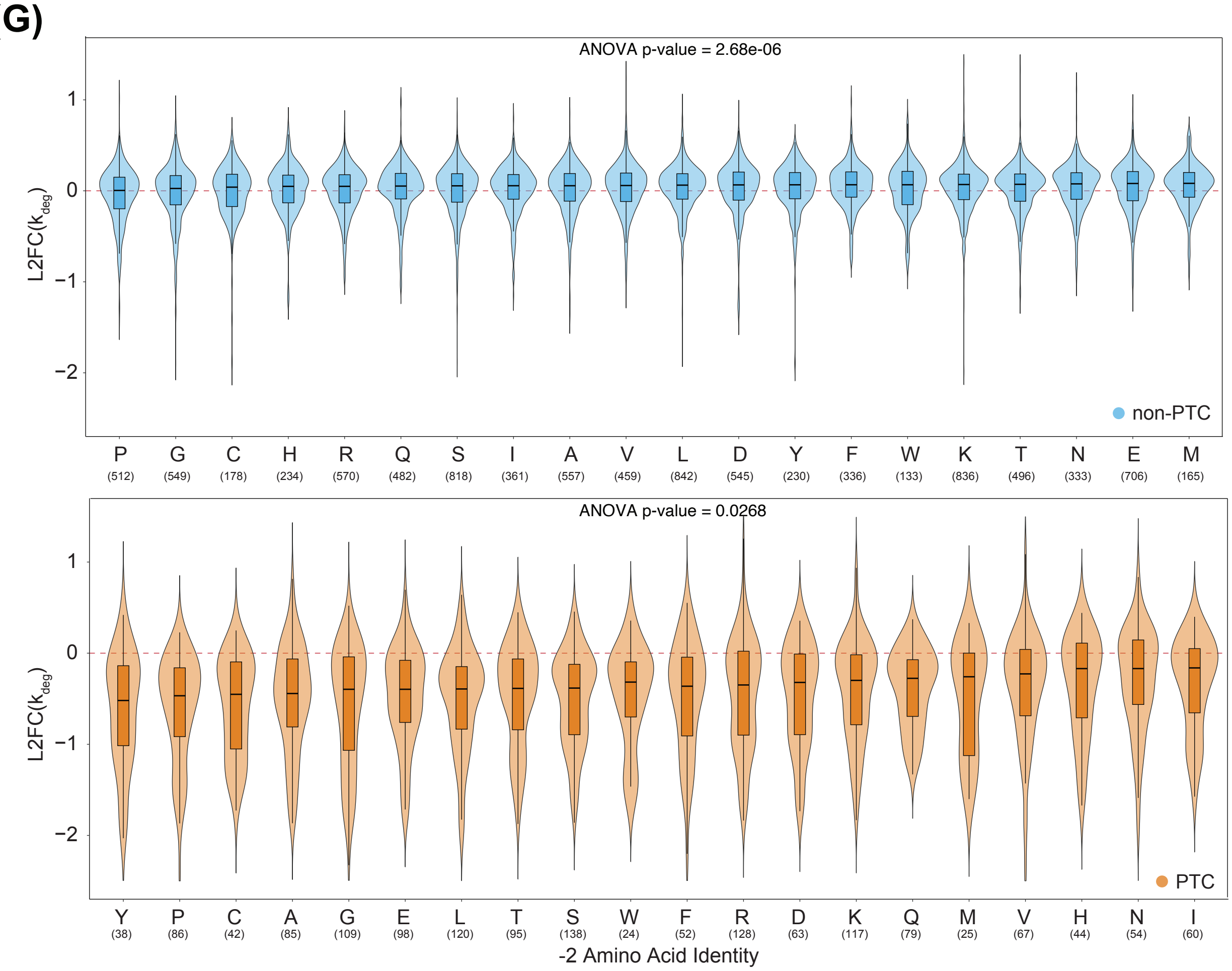
ANOVA p-value = 2.68e-06
L2FC(k_deg)
non-PTC
P (512) G (549) C (178) H (234) R (570) Q (482) S (818) I (361) A (557) V (459) L (842) D (545) Y (230) F (336) W (133) K (836) T (496) N (333) E (706) M (165)
ANOVA p-value = 0.0268
PTC
Y (38) P (86) C (42) A (85) G (109) E (98) L (120) T (95) S (138) W (24) F (52) R (128) D (63) K (117) Q (79) M (25) V (67) H (44) N (54) I (60)
-2 Amino Acid Identity

Fig. S8. Performance and results of machine learning analyses revealing key features of PTC and non-PTC isoform-specific NMD kinetics, associated with Fig. 7.
LASSO binary classification model evaluation. (**A**) Receiver operating characteristic (ROC) curves for classification of PTC and non-PTC containing transcripts as SMG1i sensitive. False positive rates (FPR) are plotted against true positive rates (TPR) for a range of cutoffs. The area under the curve (AUROC) is reported as a single overall metric. (**B**) Precision-recall curves for classification of PTC and non-PTC containing transcripts as SMG1i sensitive. The true positive rate (Recall, i.e., TPR) is plotted against the precision (1 - FDR; FDR = false discovery rate) for a range of classification cutoffs. The area under the curve (AUPRC) is reported as a single overall metric. **(C)** Scatter plot showing L2FC(k_{deg}) differences between SMG1i and DMSO treatments versus L2FC(RNA) enrichment from CLIP-UPF1 RNA affinity purification (RAP). SMG1i-responsive non-PTC and PTC isoforms are represented in blue and orange, respectively, and non-responsive isoforms are in gray. Vertical lines indicate the median of each group's distribution. Data are the same as Fig. 7C and isoforms are labeled as in Fig. 6. Top: Density plot of each group with vertical lines indicating the median of each group's distribution. **(D)** Scatter plot of L2FC(k_{deg}) versus GC-content 200 nt downstream of TC, colored as in (A). Top: Density plot for each group, with vertical lines indicating the median of each distribution. ANOVA p-value reflects statistical significance as described in Fig. 7D. **(E-G)** Violin plots showing L2FC(k_{deg}) differences between SMG1i and DMSO treatments for non-PTC (blue, top) and PTC (orange, bottom) isoforms, grouped by **(E)** TC tetranucleotide sequence, **(F)** C-terminal -1 amino acid, and **(G)** C-terminal -2 amino acid. The horizontal dashed red line represents no change in L2FC(k_{deg}) between conditions. Sample sizes for each category are shown below the plots. ANOVA p-value reflects statistical significance as described in Fig. 7D.
